# Intraparenchymal Neural Stem/Progenitor Cell Transplantation for Ischemic Stroke Animals: A Meta-Analysis and Systematic Review

**DOI:** 10.1155/2018/4826407

**Published:** 2018-10-02

**Authors:** Hailong Huang, Kun Qian, Xiaohua Han, Xin Li, Yifeng Zheng, Zhishui Chen, Xiaolin Huang, Hong Chen

**Affiliations:** ^1^Department of Rehabilitation Medicine, Tongji Hospital, Tongji Medical College, Huazhong University of Science and Technology, Wuhan 430030, China; ^2^Department of Reproductive Medicine, Tongji Hospital, Tongji Medical College, Huazhong University of Science and Technology, Wuhan 430030, China; ^3^Department of Neurosurgery, Tongji Hospital, Tongji Medical College, Huazhong University of Science and Technology, Wuhan 430030, China; ^4^Institute of Organ Transplantation, Key Laboratory of the Ministry of Health and the Ministry of Education, Tongji Hospital, Tongji Medical College, Huazhong University of Science and Technology, Wuhan 430030, China; ^5^Key Laboratory of Organ Transplantation, Ministry of Education, Wuhan 430030, China; ^6^Key Laboratory of Organ Transplantation, Ministry of Health, Wuhan 430030, China

## Abstract

Intraparenchymal transplantation of neural stem/progenitor cells (NSPCs) has been extensively investigated in animal models of ischemic stroke. However, the reported therapeutic efficacy was inconsistent among studies. To evaluate this situation, PubMed, Embase, and Web of Science databases were searched for preclinical studies using NSPC intraparenchymal transplantation in ischemic stroke animals. Data of study quality score, neurobehavioral (mNSS, rotarod test, and cylinder test) and histological (infarct volume) outcomes, cell therapy-related serious adverse events, and related cellular mechanisms were extracted for meta-analysis and systematic review. A total of 62 studies containing 73 treatment arms were included according to our criterion, with a mean quality score of 5.10 in 10. Among these studies, almost half of the studies claimed no adverse events of tumorigenesis. The finally pooled effect sizes for neurobehavioral and histological assessments were large (1.27 for mNSS, 1.63 for the rotarod test, 0.71 for the cylinder test, and 1.11 for infarct volume reduction). With further analysis, it was found that the administration time poststroke, NSPC donor species, and transplantation immunogenicity had close correlations with the degree of infarct volume reduction. The NSPC dosage delivered into the brain parenchyma was also negatively correlated with the effect of the cylinder test. Intriguingly, endogenous apoptosis inhibition and axonal regeneration played the most critical role in intraparenchymal NSPC transplantation among the cellular mechanisms. These results indicate that intraparenchymal NSPC transplantation is beneficial for neurobehavioral and histological improvement and is relatively safe for ischemic stroke animals. Therefore, intraparenchymal NSPC transplantation is a promising treatment for stroke patients.

## 1. Introduction

Ischemic stroke is one of the leading causes of death and disability around the world [1, 2]. After stroke, approximately 90% of survivors experience motor dysfunction [3], which lasts for the rest of their life and affects their daily life quality severely. However, there are few effective treatments for the ischemic stroke. Stem cell transplantation to rescue the motor function deficits poststroke has attracted a growing interest [4–6].

Among the various cell populations used for stroke cell therapy, neural stem/progenitor cells (NSPCs) and mesenchymal stem cells (MSCs) are both investigated extensively. Meta-analysis indicates that MSC therapy lies mainly in the time-dependent bystander mediators poststroke [7–9] as MSCs possess the poor potential of neural lineage differentiation. In contrast, NSPCs were regarded as a more appropriate source because of their capabilities of differentiation to neural cell phenotypes *in vitro* and *in vivo* [10, 11]. Transplanted NSPCs can migrate to peri-ischemic areas and ameliorate functional deficits [11–13]. Multiple but inconsistent mechanisms by which NSPCs enhance functional recovery were proposed, from neuroprotection [14] to neuroregeneration [15]. Similarly, the curative benefit is conflicting among studies when the following factors are involved, including donor cell states, dosage, immunogenicity, administration time after stroke onset, and immunosuppressive medicine usage. Therefore, there is a need for systematic analysis of the studies on intraparenchymal NSPC transplantation.

We conducted a meta-analysis to determine whether intraparenchymal NSPC transplantation is beneficial in preclinical studies based on neurobehavioral tests (mNSS, rotarod test, and cylinder test) and histological outcome (infarct volume). In addition, we pooled results about the cellular mechanisms and serious adverse events (SAEs). We hope that this analysis provides information for potential future clinical trials involving stem cell transplantation in stroke.

## 2. Methods

This meta-analysis was carried out following the guidelines of Preferred Reporting Items for Systematic Reviews and Meta-Analyses (PRISMA, http://www.prisma-statement.org/, Table S1 in Supplementary Materials available online) [16].

### 2.1. Search Strategy

We searched for correlative studies about neural stem/progenitor cell (NSPC) transplantation in animal models of ischemic stroke in three databases (PubMed, Embase, and Institute for Scientific Information Web of Science databases, up until July 11, 2018) by independent investigators. The search strategy was as follows: ((neural stem cells) OR (stem cell, neural) OR (neural progenitor cell) OR (neural precursor cell) OR NSPC) AND (stroke OR ischemic stroke OR brain ischemia OR brain infarction OR cerebral ischemia OR intracranial ischemia OR cerebrovascular OR middle cerebral artery OR anterior cerebral artery). The default language for all included studies was English. After studies were extracted, the titles, abstracts, and the secondary references were reviewed carefully. If controversy existed in whether the study is eligible, the study would be examined again and all investigators would discuss to reach a consensus.

### 2.2. Inclusion and Exclusion Criteria

This meta-analysis included controlled studies claiming that NSPCs and other vehicles (culture medium, PBS, or saline) were delivered intraparenchymally in ischemic stroke animals (nonhuman). For all included studies, neurobehavioral function assessments must be served as one kind of outcome indicators. We excluded studies with brain lesions other than cerebral ischemia or with cell therapy paradigm using mature cells or genetically modified cells other than those for labeling or tracing in vivo or preconditioned cell transplantation. We also excluded studies without precise animal numbers or outcome values for individual comparison.

### 2.3. Study Quality Assessment

To estimate methodological quality for all included studies, the CAMARADES (Collaborative Approach to Meta-Analysis and Review of Animal Data from Experimental Studies) checklist was applied as follows: (1) publication on a peer-reviewed journal, (2) control of temperature, (3) randomization to treatment groups, (4) allocation concealment, (5) blinded assessment of outcomes, (6) avoidance of neuroprotective anesthetics (mostly known as ketamine), (7) use of animals with relevant comorbidities, (8) sample size calculation, (9) compliance with animal welfare regulations, and (10) statement of conflict of interest. We endowed each item one point and got the total score of every study after retrieving the full text, as well as the supplementary data and secondary references.

### 2.4. Data Extraction

Besides the study quality score, we extracted the following data from each study: first author, published year, recipient species, animal models, donor species, cell characteristics (intervention time relative to stroke onset, graft sites, and cell dose), administration of immunosuppressive drugs, and sensorimotor or histological outcomes at the final time point recorded (showing the four most used data of mNSS, rotarod test, cylinder test, and infarct volume). For the safety of intraparenchymal NSPC transplantation, data of treatment-related serious adverse events (tumor/teratoma formation, seizure, infection, or death) were considered. In addition, the data of cellular treatment effects [17] (Table S2 in Supplementary Materials available online) were extracted for further analysis. The outcome values of the rotarod test were multiplied by −1 given its positive relationship with the behavioral outcome in contrast to the other three measures [7]. GetData Graph Digitizer (version 2.24) was used to quantify the mean value and standard deviation (SD) or standard error (SE) from figures, if no detailed data was referred to in the text. When all data were shown in SE, SD could be recalculated with the following formula [18]:

(1) $$\begin{array}{c} SD=\sqrt{N}\times SE, \end{array}$$

where *N* means the group size.

### 2.5. Statistical Analysis

All statistical analyses were performed using Stata (ver. 12.0, StataCorp). We evaluated the standard mean difference (SMD), 95% confidence interval (CI), and significance in a DerSimonian and Laird random-effects model by the statistics of Hedges' *g* across all studies. The therapeutic effects of intraparenchymally transplanted NSPCs in ischemic stroke were determined by obtaining mean effect sizes, with a value of <0.2 defined as a small effect, 0.2–0.8 as a medium effect, and >0.8 as a large effect [19]. If heterogeneity defined by the *I* ^2^ metric exists among different studies, with a value of 25%, 50%, and 75% considered to be low, moderate, and considerable heterogeneity, sensitivity analysis was used for heterogeneity analysis. Subgroup analysis and metaregression with the following clinical parameters were used for further evaluation.

Univariate metaregression analysis was carried out according to 12 variables, including (1) timing of NSPC intervention after stroke onset, (2) NSPC dose, (3) ischemic stroke models (transient, permanent), (4) graft sites (focal or global), (5) degree of NSPC immunogenicity (allogeneic or xenogeneic), (6) species of NSPC recipients, (7) species of NSPC donors, (8) state of donor NSPCs (pluripotent stem cell derivatives or primary cells), (9) administration of immunosuppressive drugs, (10) design in blindness, (11) randomization, and (12) study quality score. Afterwards, funnel plots were used to check the potential of publication bias for a visual impression. The data of significant publication bias was reassessed using a 2-tailed Egger regression intercept method. If publication bias existed, trim and fill analysis was used to adjust the bias and check whether the effect size affects the final results.

## 3. Results

### 3.1. Selection and Description of Involved Studies

**Figure 1:**
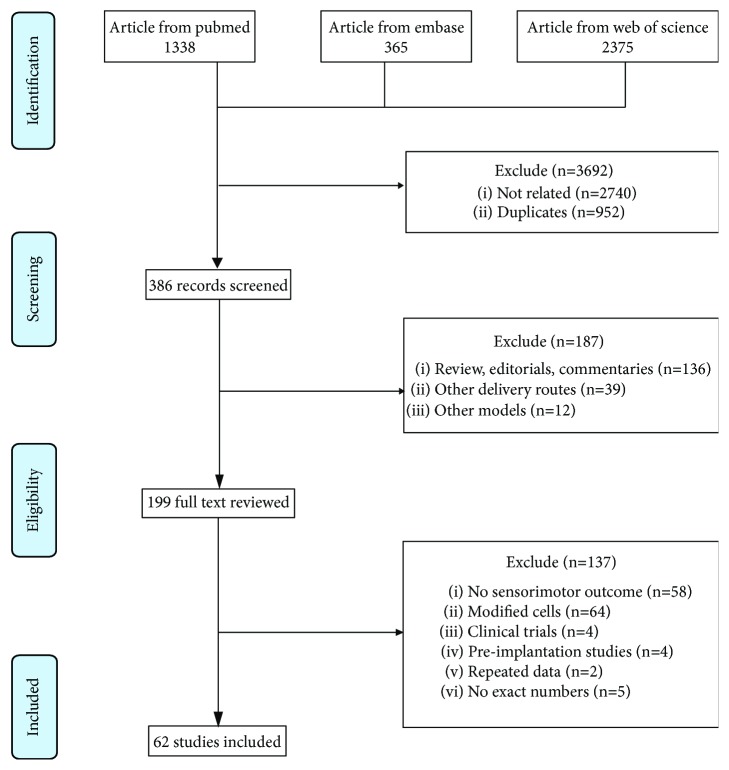
PRISMA flow diagram of including studies for this meta-analysis.

The search procedure and strategies are described in Figure 1. A total of 4078 potentially relevant studies were extracted from three databases. After excluding 3692 irrelevant studies and 187 nonstandard papers, 199 studies with full text were reviewed. According to the inclusion and exclusion criteria, 137 studies dissatisfying the eligibility criteria were excluded. Finally, 62 studies containing 73 treatment arms without duplicate data description were included in this meta-analysis (Table S3 in Supplementary Materials available online). Among these identified studies, the ischemic stroke models were made transiently (46 of 62 studies) or permanently (16 of 62 studies). Rats (40 of 62 studies) and mice (19 of 62 studies) were used as recipient species, while three studies used Mongolian gerbil [20, 21] and pig [22]. The gender of all included animals was mainly male, except for seven studies with no statements [22–28], one study using female rats [29], and one study stating nonsex difference [30]. Meanwhile, human (28 of 62 studies), rat (14 of 62 studies), and mouse (20 of 62 studies) cells were chosen as the NSPC donors. The transplanted NSPCs were either from primary cultures (45 of 73 comparisons) or from pluripotent stem cell derivatives (28 of 73 comparisons). There were also 21 studies stating the usage of immunosuppression drugs and 24 studies without it; the rest did not make a clear statement.

For SAEs related to intraparenchymal NSPC transplantation, tumor formation was most reported in 30 of the 62 studies (shown with an asterisk symbol in Table S3 in Supplementary Materials available online), but with no quantitative data.

### 3.2. Study Quality Assessment

The mean quality score across all studies was 5.10, ranging from 2 to 8 (Table S4 in Supplementary Materials available online). After being standardized by CAMARADES checklists, all the included studies were published in peer-reviewed journals and had claimed compliance with animal welfare regulations. No animals with relevant comorbidities were used for any of the studies. In addition, only thirteen studies did not describe the control of temperature, three studies had allocation concealment stressed, about 27 studies declared randomization to treatment, 39 studies described blinded assessment of outcomes, 38 studies avoided ketamine as anesthetics, and 35 studies stated conflict of interest (Table 1).

### 3.3. Meta-Analysis and Effect Size

**Figure 2:**
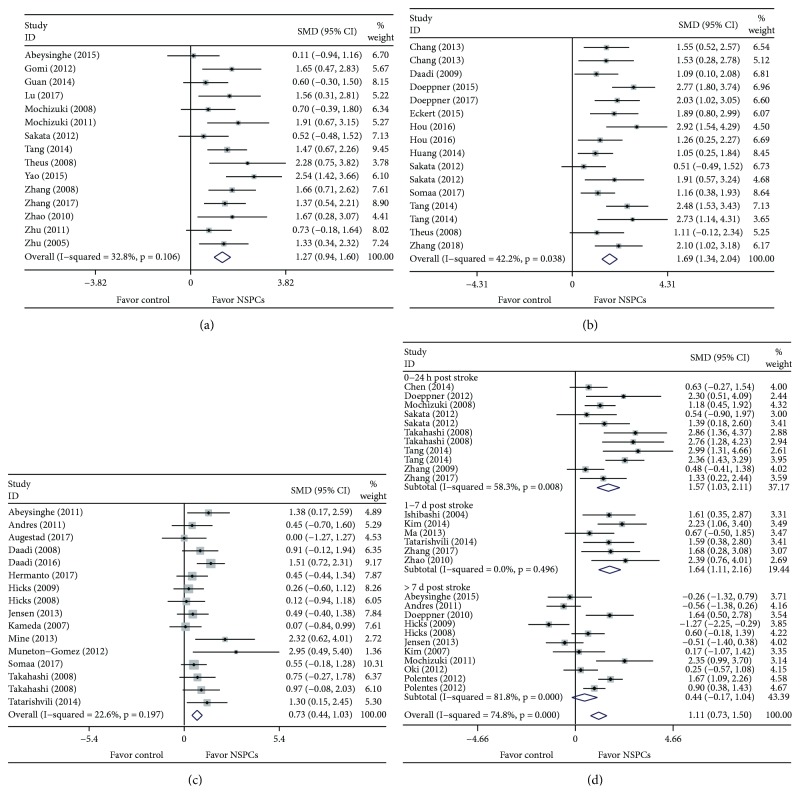
Effect sizes for intraparenchymal NPSC transplantation across related studies. Forest plot shows the mean effect sizes and 95% CI for (a) mNSS, (b) rotarod test, (c) cylinder test, and (d) infarct volume. SMD: standardized mean difference; CI: confidence interval; W: weight.

Random-effects meta-analysis was performed for four considered measurements, and the pooled effect sizes ranged from 0.73 to 2.02 (2.02 (95% CI: 1.50–2.55) for mNSS, 1.54 (95% CI: 0.92–2.15) for the rotarod test, 0.73 (95% CI: 0.44–1.03) for the cylinder test, and 1.24 (95% CI: 0.83–1.65) for infarct volume) (Figure S1 in Supplementary Materials available online). However, the heterogeneity between studies for mNSS was large (*χ* ^2^ = 84.74, *p* ≤ 0.001, *I* ^2^ = 75.2%, df = 21) (Figure S1(a) in Supplementary Materials available online). By sensitivity analysis, seven treatment arms whose effect size was greater than 3.0 were removed and the heterogeneity decreased (*χ* ^2^ = 20.82, *p* = 0.106, *I* ^2^ = 32.8%), with the corrected effect size of 1.27 (95% CI: 0.94–1.60) (Figure 2(a)). For the rotarod test, the heterogeneity among studies was big (*χ* ^2^ = 169.97, *p* ≤ 0.001, *I* ^2^ = 87.1%, df = 22) (Figure S1(b) in Supplementary Materials available online). When sensitivity analysis was performed, the heterogeneity went down (*χ* ^2^ = 25.97, *p* = 0.038, *I* ^2^ = 42.2%) (Figure 2(b)) after three treatment arms with effect size greater than 3.0 and four other inadaptable comparisons were removed (Table S5 in Supplementary Materials available online). The corrected effect size was 1.69 (95% CI: 1.34–2.04) (Figure 2(b)). The heterogeneity among studies for the cylinder test was moderate (*χ* ^2^ = 19.38, *p* = 0.197, *I* ^2^ = 22.6%, df = 15) (Figure S1(c) in Supplementary Materials available online; Figure 2(c)). As for the infarct volume, the heterogeneity among studies was high (*χ* ^2^ = 135.19, *p* ≤ 0.001, *I* ^2^ = 77.1%, df = 31) (Figure S1(d) in Supplementary Materials available online). After four inadaptable comparisons were removed by sensitivity analysis, the heterogeneity was slightly altered (*χ* ^2^ = 107.27, *p* ≤ 0.001, *I* ^2^ = 74.8%), with the corrected effect size of 1.11 (95% CI: 0.73–1.50) (Figure 2(d)).

Effect sizes for cellular mechanisms related to NSPC transplantation in ischemic stroke animals, including apoptosis inhibition, immunomodulation, neurotrophic factor release, angiogenesis, neurogenesis, gliosis reduction, white matter function, enzyme supplementation, and axonal function, were evaluated (Table 2). Among these mechanisms, apoptosis reduction and axonal function were the most obvious effects following NSPC intraparenchymal transplantation (Table 2).

### 3.4. Subgroup Analysis and Metaregression Analysis

**Figure 3:**
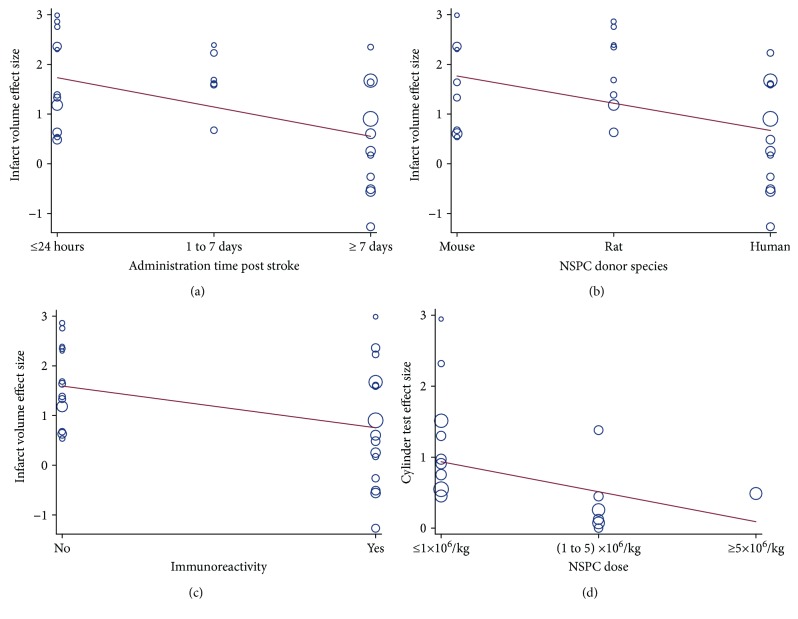
Metaregression analysis for related variables and effect sizes of infarct volume reduction and cylinder test. (a) An earlier intraparenchymal NSPC transplantation was associated with a smaller tissue loss (Adj *R* ^2^ = 26.92%, *p* = 0.008). (b) Rodent cell donors were associated with greater residual tissue (Adj *R* ^2^ = 20.00%, *p* = 0.030). (c) Allograft benefited the infarct volume reduction more (Adj *R* ^2^ = 15.24%, *p* = 0.044). (d) A relatively lower NSPC dosage was associated with larger cylinder test performance (Adj *R* ^2^ = 100.00%, *p* = 0.063). Values for effect sizes are Hedges' *g*.

Following the effect size evaluation and sensitivity analysis, the heterogeneity in infarct volume effect size was still detected. Then, subgroup analysis and metaregression analysis based on the 12 clinically related parameters were chosen to analyze their contribution to statistical heterogeneity for infarct volume effect size. We found that administration time poststroke was negatively related to the infarct volume effect size (Adj *R* ^2^ = 26.92%, *p* = 0.008), with an earlier NSPC transplantation and a bigger effect size (Table 3, Figure 3(a)). NSPC donor species also had a close correlation with infarct volume effect size (Adj *R* ^2^ = 20.00%, *p* = 0.025), with mouse donors having more infarct volume reduction, indicating a preference for homologous transplantation (Table 3, Figure 3(b)). In addition, the transplantation immunity was correlated with the infarct volume reduction, with allograft indicating more reserved tissue (Adj *R* ^2^ = 15.24%, *p* = 0.044) (Figure 3(c)). Meanwhile, for the cylinder test effect size, the NSPC dosage was discovered to be a negative correlative variable, with low dose for a better behavioral function (Adj *R* ^2^ = 100.00%, *p* = 0.063) (Figure 3(d)).

### 3.5. Publication Bias

**Figure 4:**
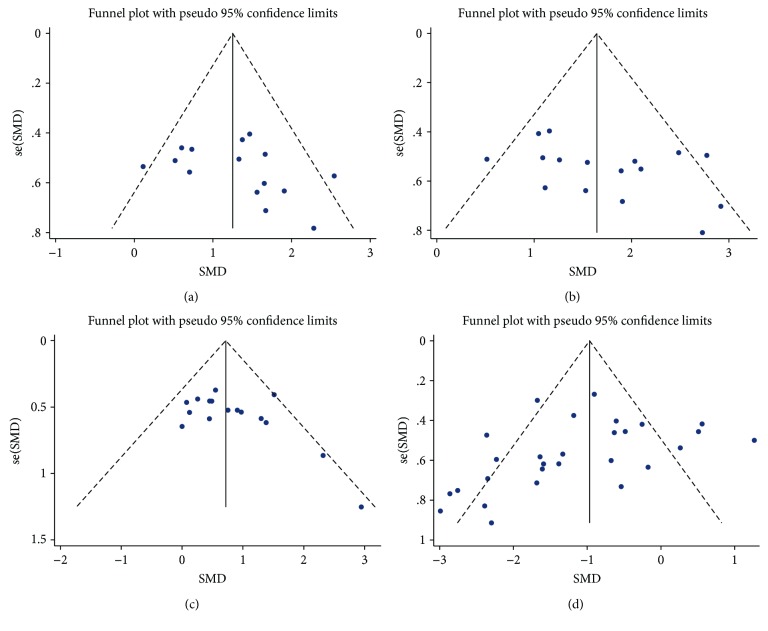
Funnel plots for publication bias of (a) mNSS, (b) rotarod test, (c) cylinder test, and (d) infarct volume. SMD: standardized mean difference.

From the funnel plots (Figure 4), an approximately symmetrical distribution for mNSS and infarct volume was visualized, which means no publication bias. This was further confirmed by the Egger test in which the relative *p* values were 0.185 and 0.110 for mNSS and infarct volume, respectively, both of which exceeded 0.1. Although publication bias existed for the rotarod test and the cylinder test, with *p* values related to the Egger test equal to 0.094 and 0.073, respectively, these two recalibrated effect sizes were still larger than 1 (1.63 for the rotarod test and 0.71 for the cylinder test) after trim and fill analysis.

## 4. Discussion

This meta-analysis includes 62 controlled animal studies published from year 2004 to 2018. We found that intraparenchymal NSPC transplantation benefits neurobehavioral (mNSS, rotarod test, and cylinder test) and histological (infarct volume) outcomes poststroke with large effect sizes. The effect size of infarct volume reduction is correlated closely with administration time poststroke, NSPC donor species, and transplantation immunogenicity. The cylinder test effect size is correlated with NSPC donor dosage. No correlation was found between neurobehavioral or histological changes and other variables, such as stroke models, graft sites, species of NSPC recipients, state of donor NSPCs, immunosuppressive drugs, blinding and randomization design, and study quality. The main mechanism by which intraparenchymal NSPC transplantation benefits ischemic stroke appears to lie in apoptosis inhibition and axonal function.

According to the STAIR guidance for methodological quality estimation [31], a mean quality score of 5.10 in this meta-analysis is higher than previous reports [32, 33]. However, the methodological quality of animal studies should be improved because the overestimation effect of low-quality studies potentially influences the results [34, 35]. After further analysis, we could not find direct proof in study quality score and neurobehavioral or histological effect size as was described in a similar study [36], and publication bias existed in neurobehavioral indices (rotarod and cylinder test), both of which encourage future research with rigorous design.

In this meta-analysis, the overall effect sizes for four considered indices are big, which would suggest an improvement in neurobehavioral and histological outcomes in adult ischemic stroke animals after intraparenchymal NSPC transplantation. However, because of the existence of heterogeneity among studies for mNSS, rotarod test, and infarct volume, the output results should be interpreted with care. It also should be mentioned that there were several comparisons with an effect size more than 3.0, which contributes to the heterogeneity significantly [7, 17, 33]. After examining these studies, we found that there are some defects in the experimental design, including no blinding, no randomization, nonstatistical animal numbers, low-quality study, nonnormative procedure for assessments without pretraining, and low reliability for gained results. By sensitivity analysis to exclude inadequate comparisons, we obtained more homogenous data to draw a reliable conclusion for effect sizes of mNSS (1.27 SMD), rotarod test (1.63 SMD), and infarct volume.

The parameters used in this meta-analysis, including NSPC administration time, dosage, donor species, and transplantation immunogenicity, may be useful for future clinical application of stem cell therapy. Our metaregression analysis reveals that an earlier NSPC transplantation, that is, at the acute or subacute stage (within a period of 7 days), results in a greater reduction in infarct volume. This is partially consistent with the conclusions of a recent meta-analysis [33]. Compared with the late NSPC transplantation, the early delivery may spare secondary damage to the brain tissue and preserve neural circuits more by neuroprotection effects [37, 38]. Interestingly, we found that a relatively low amount of NSPCs for intraparenchymal transplantation could improve the cylinder test more than the high-dose groups. The effective cell dose is less than 1 × 10^6^ cells/kg. This appears to contradict the perceived understanding that a better neurobehavioral outcome results from a higher cell dose. A relatively low dose of NSPCs delivered intraparenchymally could migrate to the lesion area directly without exacerbating the ischemic brain injury by a high dose (volume) of grafted cells and tumor formation. Our subgroup meta-analysis and metaregression analysis also suggest that NSPCs from mouse donors contribute to a larger effect size of infarct volume reduction than those from rats and humans. And a more favorable lesion reduction effect is achieved through allogeneic transplantation rather than xenotransplantation, that is, rodent NSPCs to rodents, consistent with the results of Chen et al. [33] and Yousefifard et al. [39] in NSPC transplantation for spinal cord injury. This is likely related to the immunological reactions. For human NSPC grafts, the transplantation paradigm of mouse-mouse was within species, with low immune response to host tissue or cell grafts [40]. Finally, another factor that determines clinical translation is tumorigenesis. In our meta-analysis, about half of the included studies have claimed no malignant neoplasm formation from cell grafts. Since most of the NSPCs in the included studies are derived from pluripotent stem cells, this suggests that the cells used for transplantation are a reasonably appropriate source for stroke therapy [41].

The mechanisms underlying stem cell transplantation for ischemic stroke are not clear. Meta-analysis on MSC transplantation for stroke animal studies [7, 36, 42] suggests the involvement of apoptosis inhibition, immunomodulation, neurotrophic factors, angiogenesis, neurogenesis, gliosis reduction, white matter function, enzyme supplementation, and axonal function. We found that apoptosis inhibition is the main contributor to neurobehavioral and histological improvement in intraparenchymal NSPC transplantation. Axonal function also has a significant role in behavioral and histological amelioration. Thus, neuroprotection and cell replacement underlie the therapeutic effect of intraparenchymal NSPC transplantation in ischemic stroke animals.

## 5. Conclusions

This study suggests that intraparenchymal delivery of NSPCs is a promising candidate for ischemic stroke therapy, improving the functional deficits and histopathology in animal models of ischemic stroke. The possible cellular mechanisms associated with intraparenchymal NSPC transplantation include apoptosis inhibition and axonal function.

## Figures and Tables

**Table 1:** Distribution of included studies according to CAMARADES checklists.

Criteria	Number of studies	Per (%)
Publication on a peer-reviewed journal	62	100
Control of temperature	49	79.0
Randomization to a treatment group	27	43.5
Allocation concealment	3	4.8
Blinded assessment of outcome	39	62.9
Avoidance of neuroprotective anesthetics	38	61.3
Use of animal with relevant comorbidities	0	0
Sample size calculation	0	0
Compliance with animal welfare regulations	62	100
Statement of conflict of interest	35	56.5

**Table 2:** Effect sizes for cellular treatment effects.

Treatment effects	Effect size	95% CI	Treatment arms
Apoptosis inhibition	3.09	1.94–4.25	13
Immunomodulation	1.47	0.34–2.61	13
Growth factors (BDNF)	1.37	0.57–2.17	14
Growth factors (VEGF)	1.15	0.47–1.84	13
Angiogenesis	1.18	0.42–1.94	11
Neurogenesis	1.23	0.40–2.07	13
Gliosis reduction	2.35	0.22–4.49	5
White matter function	1.00	0.40–1.61	3
Enzyme supplementation	0.81	−0.36–1.97	6
Axonal function	2.47	1.36–3.57	1

**Table 3:** Subgroup meta-analysis and metaregression of variants correlated with infarct volume reduction.

Clinical variants	Effect size (95% CI)	*I* ^2^, *p* value	df	Univariate analysis (Adj *R* ^2^, *p*)
Administration time poststroke				26.92%, 0.008
≤24 h	1.57 (1.03–2.21)	58.3%, 0.008	10	
1–7 d	1.64 (1.11–2.16)	0.0%, 0.496	5	
≥7 d	0.44 (−0.17–1.04)	81.8%, ≤0.001	10	
Cell dosage				1.27%, 0.261
≤1 × 10^6^ cells/kg	1.45 (0.99–1.91)	57.4%, 0.012	9	
1–5 × 10^6^ cells/kg	0.82 (0.06–1.59)	83.1%, ≤0.001	10	
>5 × 10^6^ cells/kg	0.99 (0.25–1.73)	61.5%, 0.016	6	
Animal model				0.79%, 0.280
Transient	1.24 (0.82–1.67)	68.9%, ≤0.001	19	
Permanent	0.78 (−0.07–1.64)	82.7%, ≤0.001	7	
Graft sites				
Global	0.24 (−0.59–1.08)	40.1%, 0.196	1	2.06%, 0.201
Focal	1.19 (0.78–1.60)	75.2%, ≤0.001	25	
Cell donor species				20.00%, 0.025
Mouse	1.46 (0.84–2.08)	54.7%, 0.031	7	
Rat	1.73 (1.15–2.31)	45.7%, 0.075	7	
Human	0.51 (−0.08–1.10)	81.0%, ≤0.001	11	
Cell recipient species				−4.82%, 0.761
Mouse	0.98 (0.40–1.56)	31.2%, 0.201	5	
Rat	1.12 (0.64–1.60)	79.8%, ≤0.001	20	
Others	1.61 (0.35–2.87)		0	
Immunoreactivity				15.24%, 0.044
No	1.52 (1.11–1.93)	30.9%, 0.136	12	
Yes	0.76 (0.21–1.31)	82.1%, ≤0.001	14	
State of donor cells				5.14%, 0.115
PSC-NSPCs	0.63 (−0.13–1.38)	84.1%, ≤0.001	7	
WT-NSPCs	1.33 (0.88–1.78)	68.2%, ≤0.001	19	
Immunosuppression drugs				−3.64%, 0.657
No	1.17 (0.22–2.12)	78.6%, ≤0.001	6	
Yes	0.95 (0.32–1.58)	83.2%, ≤0.001	11	
Unknown	1.28 (0.82–1.74)	26.9%, 0.205	8	
Blinding				−5.59%, 0.758
No	1.32 (0.17–2.46)	85.8%, ≤0.001	6	
Yes	1.06 (0.67–1.46)	69.1%, ≤0.001	20	
Randomization				−3.47%, 0.483
No	1.33 (0.61–2.05)	77.1%, ≤0.001	10	
Yes	0.99 (0.53–1.46)	74.6%, ≤0.001	16	

## Supplementary Materials

Supplementary 1 Figure S1: effect sizes of all included studies for (a) mNSS, (b) rotarod test, (c) cylinder test, and (d) infarct volume.

Supplementary 2 Table S1: PRISMA 2009 checklist.

Supplementary 3 Table S2: extracted data associated with cellular mechanisms of NSPC intraparenchymal transplantation in ischemic stroke animals.

Supplementary 4 Table S3: characteristics for all included studies.

Supplementary 5 Table S4: study quality assessment referred to the CAMARADES checklists.

Supplementary 6 Table S5: reasons for excluding related comparisons.

## References

[1] Feigin V. L., Roth G. A., Naghavi M. (2016). Global burden of stroke and risk factors in 188 countries, during 1990–2013: a systematic analysis for the Global Burden of Disease Study 2013. *The Lancet Neurology*.

[2] Liu L., Wang D., Wong K. S. L., Wang Y. (2011). Stroke and stroke care in China: huge burden, significant workload, and a national priority. *Stroke*.

[3] Hesse S., Werner C. (2003). Poststroke motor dysfunction and spasticity: novel pharmacological and physical treatment strategies. *CNS Drugs*.

[4] Aked J., Delavaran H., Lindvall O., Norrving B., Kokaia Z., Lindgren A. (2017). Attitudes to stem cell therapy among ischemic stroke survivors in the Lund Stroke Recovery Study. *Stem Cells and Development*.

[5] Lindvall O., Kokaia Z. (2010). Stem cells in human neurodegenerative disorders—time for clinical translation?. *The Journal of Clinical Investigation*.

[6] Dimyan M. A., Cohen L. G. (2011). Neuroplasticity in the context of motor rehabilitation after stroke. *Nature Reviews Neurology*.

[7] Vu Q., Xie K., Eckert M., Zhao W., Cramer S. C. (2014). Meta-analysis of preclinical studies of mesenchymal stromal cells for ischemic stroke. *Neurology*.

[8] Lalu M. M., McIntyre L., Pugliese C. (2012). Safety of cell therapy with mesenchymal stromal cells (SafeCell): a systematic review and meta-analysis of clinical trials. *PLoS One*.

[9] Li Y., Chen J., Wang L., Lu M., Chopp M. (2001). Treatment of stroke in rat with intracarotid administration of marrow stromal cells. *Neurology*.

[10] Doe C. Q. (2008). Neural stem cells: balancing self-renewal with differentiation. *Development*.

[11] Daadi M. M., Maag A. L., Steinberg G. K. (2008). Adherent self-renewable human embryonic stem cell-derived neural stem cell line: functional engraftment in experimental stroke model. *PLoS One*.

[12] Modo M., Stroemer R. P., Tang E., Patel S., Hodges H. (2002). Effects of implantation site of stem cell grafts on behavioral recovery from stroke damage. *Stroke*.

[13] Bacigaluppi M., Pluchino S., Jametti L. P. (2009). Delayed post-ischaemic neuroprotection following systemic neural stem cell transplantation involves multiple mechanisms. *Brain*.

[14] Giusto E., Donega M., Cossetti C., Pluchino S. (2014). Neuro-immune interactions of neural stem cell transplants: from animal disease models to human trials. *Experimental Neurology*.

[15] Borlongan C. V. (2016). Age of PISCES: stem-cell clinical trials in stroke. *Lancet*.

[16] Moher D., Liberati A., Tetzlaff J., Altman D. G., The PRISMA Group (2009). Preferred reporting items for systematic reviews and meta-analyses: the PRISMA statement. *PLoS Medicine*.

[17] Janowski M., Walczak P., Date I. (2010). Intravenous route of cell delivery for treatment of neurological disorders: a meta-analysis of preclinical results. *Stem Cells and Development*.

[18] Gardner M. J., Altman D. G. (1986). Confidence intervals rather than P values: estimation rather than hypothesis testing. *British Medical Journal (Clinical Research Ed.)*.

[19] Thomas J. R., Salazar W., Landers D. M. (1991). What is missing in p less than. 05? Effect size. *Research Quarterly for Exercise and Sport*.

[20] Ishibashi S., Sakaguchi M., Kuroiwa T. (2004). Human neural stem/progenitor cells, expanded in long-term neurosphere culture, promote functional recovery after focal ischemia in Mongolian gerbils. *Journal of Neuroscience Research*.

[21] Yamane J., Ishibashi S., Sakaguchi M. (2011). Transplantation of human neural stem/progenitor cells overexpressing galectin-1 improves functional recovery from focal brain ischemia in the Mongolian gerbil. *Molecular Brain*.

[22] Lau V. W., Platt S. R., Grace H. E., Baker E. W., West F. D. (2018). Human iNPC therapy leads to improvement in functional neurologic outcomes in a pig ischemic stroke model. *Brain and Behavior*.

[23] Chen L., Qiu R., Li L. (2014). The role of exogenous neural stem cells transplantation in cerebral ischemic stroke. *Journal of Biomedical Nanotechnology*.

[24] Gomi M., Takagi Y., Morizane A. (2012). Functional recovery of the murine brain ischemia model using human induced pluripotent stem cell-derived telencephalic progenitors. *Brain Research*.

[25] Mohamad O., Drury-Stewart D., Song M. (2013). Vector-free and transgene-free human iPS cells differentiate into functional neurons and enhance functional recovery after ischemic stroke in mice. *PLoS One*.

[26] Oki K., Tatarishvili J., Wood J. (2012). Human-induced pluripotent stem cells form functional neurons and improve recovery after grafting in stroke-damaged brain. *Stem Cells*.

[27] Somaa F. A., Wang T. Y., Niclis J. C. (2017). Peptide-based scaffolds support human cortical progenitor graft integration to reduce atrophy and promote functional repair in a model of stroke. *Cell Reports*.

[28] Zhang F., Duan X., Lu L. (2017). In vivo long-term tracking of neural stem cells transplanted into an acute ischemic stroke model with reporter gene-based bimodal MR and optical imaging. *Cell Transplant*.

[29] Zhao Y., Yao S. T., Wang S. J. (2010). Neural stem cell transplantation in the hippocampus of rats with cerebral ischemia/reperfusion injury activation of the phosphatidylinositol-3 kinase/Akt pathway and increased brain-derived neurotrophic factor expression. *Neural Regeneration Research*.

[30] Hou B., Ma J., Guo X. (2017). Exogenous neural stem cells transplantation as a potential therapy for photothrombotic ischemia stroke in Kunming mice model. *Molecular Neurobiology*.

[31] Fisher M., Feuerstein G., Howells D. W. (2009). Update of the stroke therapy academic industry roundtable preclinical recommendations. *Stroke*.

[32] Lees J. S., Sena E. S., Egan K. J. (2012). Stem cell-based therapy for experimental stroke: a systematic review and meta-analysis. *International Journal of Stroke*.

[33] Chen L., Zhang G., Gu Y., Guo X. (2016). Meta-analysis and systematic review of neural stem cells therapy for experimental ischemia stroke in preclinical studies. *Scientific Reports*.

[34] Macleod M. R., O'Collins T., Howells D. W., Donnan G. A. (2004). Pooling of animal experimental data reveals influence of study design and publication bias. *Stroke*.

[35] Zhang Z., Xu X., Ni H. (2013). Small studies may overestimate the effect sizes in critical care meta-analyses: a meta-epidemiological study. *Critical Care*.

[36] Hu Y., Liu N., Zhang P. (2016). Preclinical studies of stem cell transplantation in intracerebral hemorrhage: a systemic review and meta-analysis. *Molecular Neurobiology*.

[37] Sakata H., Niizuma K., Yoshioka H. (2012). Minocycline-preconditioned neural stem cells enhance neuroprotection after ischemic stroke in rats. *The Journal of Neuroscience: The Official Journal of the Society for Neuroscience*.

[38] Eckert A., Huang L., Gonzalez R., Kim H. S., Hamblin M. H., Lee J. P. (2015). Bystander effect fuels human induced pluripotent stem cell-derived neural stem cells to quickly attenuate early stage neurological deficits after stroke. *Stem Cells Translational Medicine*.

[39] Yousefifard M., Rahimi-Movaghar V., Nasirinezhad F. (2016). Neural stem/progenitor cell transplantation for spinal cord injury treatment; a systematic review and meta-analysis. *Neuroscience*.

[40] Warfvinge K., Schwartz P. H., Kiilgaard J. F. (2011). Xenotransplantation of human neural progenitor cells to the subretinal space of nonimmunosuppressed pigs. *Journal of Transplantation*.

[41] Mack G. S. (2011). ReNeuron and StemCells get green light for neural stem cell trials. *Nature Biotechnology*.

[42] Wu Q., Wang Y., Demaerschalk B. M., Ghimire S., Wellik K. E., Qu W. (2017). Bone marrow stromal cell therapy for ischemic stroke: a meta-analysis of randomized control animal trials. *International Journal of Stroke*.

