# Supplementary material for: Intraparenchymal Neural Stem/Progenitor Cell Transplantation for Ischemic Stroke Animals: A Meta-Analysis and Systematic Review
Source: Stem Cells Int. 2018 Oct 2;2018:4826407. doi: 10.1155/2018/4826407 (PMC6189667; doi:10.1155/2018/4826407)
Supplement: Supplementary 1 — Figure S1: effect sizes of all included studies for (a) mNSS, (b) rotarod test, (c) cylinder test, and (d) infarct volume. [file 4826407.f1.docx]

**
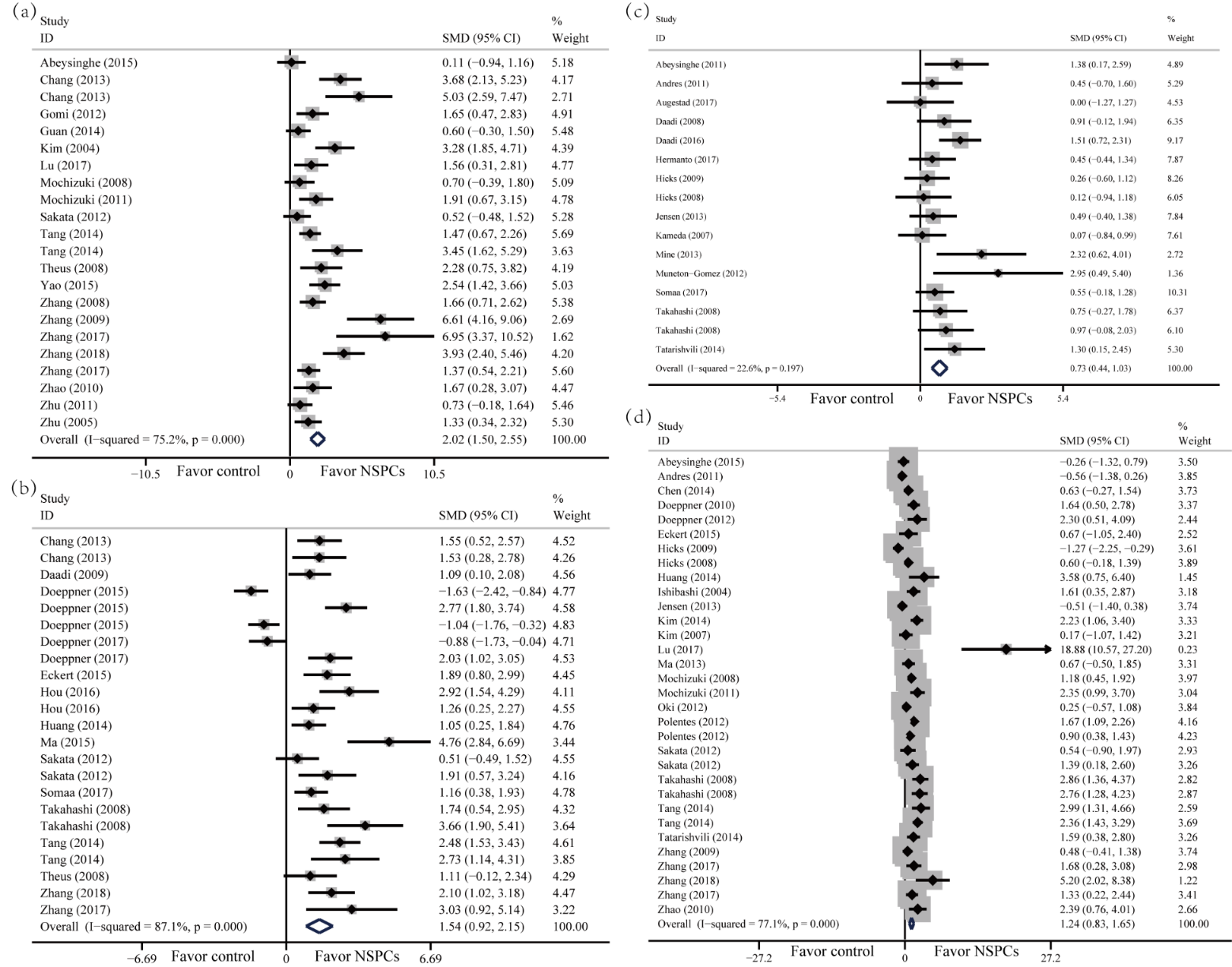
**

Figure S1. Effect sizes of all included studies for (a) mNSS, (b) rotarod test, (c) cylinder test, and (d) infarct volume.
