# Supplementary material for: Intraparenchymal Neural Stem/Progenitor Cell Transplantation for Ischemic Stroke Animals: A Meta-Analysis and Systematic Review
Source: Stem Cells Int. 2018 Oct 2;2018:4826407. doi: 10.1155/2018/4826407 (PMC6189667; doi:10.1155/2018/4826407)
Supplement: Supplementary 3 — Table S2: extracted data associated with cellular mechanisms of NSPC intraparenchymal transplantation in ischemic stroke animals. [file 4826407.f3.docx]

Table S2. Extracted data associated with cellular mechanisms of NSPC intra-parenchymal transplantation in ischemic stroke animals

| Mechanisms | Extracted indicators |
| --- | --- |
| Apoptosis inhibition | TUNEL |
| Immunomodulation | Iba1, ED1, CD11b |
| Growth factors-BDNF | BDNF |
| Growth factors-VEGF | VEGF |
| Angiogenesis | BrdU/CD31, BrdU/Col IV, blood vessel density |
| Neurogenesis | Endogenous BrdU, BrdU/DCX, BrdU/NeuN |
| Gliosis reduction | Endogenous GFAP |
| White matter function | Corpus callosum thickness |
| Enzyme supplementation | MMP-9 |
| Axonal function | Axonal density |
