# Supplementary material for: Intraparenchymal Neural Stem/Progenitor Cell Transplantation for Ischemic Stroke Animals: A Meta-Analysis and Systematic Review
Source: Stem Cells Int. 2018 Oct 2;2018:4826407. doi: 10.1155/2018/4826407 (PMC6189667; doi:10.1155/2018/4826407)
Supplement: Supplementary 4 — Table S3: characteristics for all included studies. [file 4826407.f4.docx]

Table S3. Characteristics for all included studies

| Study ID | Species/gender | Ischemic model | Donor cells | Administration X post stroke | | | Immunosuppressant | Treatment arms | Sensorimotor/histological assessments extracted |
| --- | --- | --- | --- | --- | --- | --- | --- | --- | --- |
|  |  |  |  | Time | Sites | Cell dose, cells/Kg |  |  |  |
| Abeysinghe 2015 [1] | Wistar rats/Male | ET-1 induced tMCAO | Human fetal NSC | 7 d | ipsi-cortex and striatum | (22.2-26.7)×10^5^ | Yes | 1 | Cylinder test; mNSS; infarct volume |
| Andres 2011 [2] | Nude rats/Male | Distal pMCAO and bilateral CCAO (30 min) | Human fetal NPC | 7 d | ipsi-cortex | 13×10^5^ | No | 1 | Cylinder test; infarct volume |
| Augestad 2017 [3] | SD rats/Male | tMCAO (60 min) | Human ESC-NSC | 2 wks | ipsi-globus pallidus | (11-13)×10^5^ | No | 1 | Cylinder test |
| Cai 2015 [4] | SD rats/Male | tMCAO (60 min) | Adult rat NSC | 12 d | ipsi-hippocampus | 4×10^5^ | Unknown | 1 |  |
| Chang 2013 [5] * | SD rats/Male | tMCAO (90 min) | Human iPSC-NPC | 7 d | contra-striatum | (6.7-7.4)×10^5^ | Yes | 1 | Rotarod test; mNSS |
| Chang 2013 [6] * | SD rats/Male | tMCAO (90 min) | Human ESC-NPC | 7 d | contra-striatum | (6.7-7.4)×10^5^ | Yes | 1 | Rotarod test; mNSS |
| Chau 2014 [7] * | Wistar rats | pMCAO | Mouse iPSC-NPC | 7 d | peri-infarct | ＞133×10^5^ | No | 1 |  |
| Chau 2017[8] * | C57BL/6 mice/Male | Distal pMCAO and bilateral CCAO (7 min) | Mouse iPSC-NPC | 7 d | peri-infarct | (36-40)×10^5^ | Unknown | 2 |  |
|  |  |  |  |  |  | (108-120)×10^5^ |  |  |  |
| Chen 2014 [9] | SD rats | tMCAO (90 min) | Fetal rat NSC | 24 hrs | ipsi-striatum | 3.2×10^5^ | No | 1 | Infarct volume |
| Daadi 2016 [10] | SD rats/Male | tMCAO (65 min) | Human ESC-NSC | 7 d | ipsi-striatum | (6.5-7.3)×10^5^ | Yes | 1 | Cylinder test |
| Daadi 2009 [11] | SD rats/Male | tMCAO (90 min) | Fetal rat NSC | 2 wks | ipsi-cortex and striatum | (3.2-3.6)×10^5^ | Yes | 1 | Rotarod test |
| Daadi 2008 [12] * | SD rats/Male | tMCAO (90 min) | Human ESC-NSC | 7 d | ipsi-striatum | (3.2-3.6)×10^5^ | Yes | 1 | Cylinder test |
| Doeppner 2010 [13] | C57BL/6 mice/Male | tMCAO (30 min) | Adult mouse NPC | 7 d | ipsi-striatum | (185-200)×10^5^ | No | 1 | Infarct volume |
| Doeppner 2012 [14] | C57BL/6 mice/Male | tMCAO (45 min) | Adult mouse NPC | 6 hrs | ipsi-striatum | (185-200)×10^5^ | No | 1 | infarct volume |
| Doeppner 2015 [15] | C57BL/6 mice/Male | tMCAO (45 min) | Adult mouse NPC | 6 hrs | ipsi-striatum | (185-200)×10^5^ | No | 3 | Rotarod test |
|  |  |  |  |  | contra-striatum | (185-200)×10^5^ |  |  |  |
|  |  |  |  |  | ipsi-cortex | (37-40)×10^5^ |  |  |  |
| Doeppner 2017 [16] | C57BL/6 mice/Male | tMCAO (60 min) or with another 10 min reperfusion | Fetal mouse NPC | 6 hrs | ipsilateral | (192-217)×10^5^ | No | 2 | Rotarod test |
| Drury-Stewart 2013 [17] * | C57BL/6 mice/Male | Distal pMCAO and bilateral CCAO (7 min) | Human ESC-NPC | 7 d | ipsi-cortex | 80×10^5^ | No | 1 |  |
| Eckert 2015 [18] * | C57BL/6 mice/Male | tMCAO (60 min) | Human iPSC-NSC | 24 hrs | ipsi-hippocampus | (38-42)×10^5^ | No | 1 | Rotarod test; infarct volume |
| Fujimoto 2012 [19] * | C57BL/6 mice/Male | tMCAO (45 min) | Mouse ESC-NPC | 7 d | ipsi-striatum | (67-80)×10^5^ | Unknown | 2 |  |
| Gomi 2012 [20] * | C57BL/6 mice | tMCAO (30 min) | Human iPSC-NPC | 7 d | ipsi-striatum | ＞40×10^5^ | No | 1 | mNSS |
| Guan 2014 [21] * | Wistar rats/Male | tMCAO (120 min) | Mouse ESC-NPC | 2 d | ipsi-striatum | 16.7×10^5^ | Yes | 1 | mNSS |
| Hermanto 2017 [22] | Wistar rats/Male | tMCAO (90 min) | Human iPSC-NPC | 7 d | ipsi-cortex | (1.8-2)×10^5^ | Yes | 1 | Cylinder test |
| Hicks 2009 [23] * | Wistar rats/Male | Distal pMCAO and bilateral CCAO (60 min) | Human ESC-NPC | 7 d | ipsi-cortex | (27-29)×10^5^ | Yes | 1 | Cylinder test; infarct volume |
| Hicks 2008 [24] | SD rats/Male | ET-1 induced tMCAO | Adult mouse NSC | 7 d | ipsi-cortex and striatum | (23-27)×10^5^ | Yes | 1 | Cylinder test; infarct volume |
| Hou 2016 [25] | Kunming mice/Non-sex | Photothrombotic ischemia stroke | Neonatal mouse NSC | 3 d | ipsi-cortex | (100-114)×10^5^ | No | 2 | Rotarod test |
| Huang 2014 [26] * | C57BL/6 mice/Male | tMCAO (60 min) | Human fetal NSC | 24 hrs | ipsi-hippocampus | (38-42)×10^5^ | No | 1 | Rotarod test; infarct volume |
| Ishibashi 2004 [27] | Mongolian gerbil/Male | Unilateral CCAO for 10 min | Human fetal NSPC | 4 d | ipsi-caudate nucleus | (70-83)×10^5^ | Yes | 1 | Infarct volume |
| Jensen 2013 [28] * | Wistar rats/ Male | tMCAO (30 min) | Human iPSC-NSC | 7 d | ipsi-striatum | (77-91)×10^5^ | Yes | 1 | Cylinder test; infarct volume |
| Kameda 2007 [29] | Wistar rats/ Male | tMCAO (90 min) | Adult rat NSC | 3 hrs | peri-infarct | (11-12)×10^5^ | No | 1 | Cylinder test |
| Kim 2007 [30] * | SD rats/Male | tMCAO (120 min) | Human ESC-NPC | 7 d | ipsi-cortex | (15-18)×10^5^ | No | 1 | Infarct volume |
| Kim 2014 [31] * | SD rats/Male | pMCAO | Human ESC-NPC | 2 d | ipsi-striatum | (17-20)×10^5^ | Yes | 1 | mNSS; infarct volume |
| Lau 2018 [32] | Landrace mix pigs | pMCAO | Human iPSC-NPC | 5 d | ipsi-cortex | (0.63-0.77)×10^5^ | Unknown | 1 |  |
| Lu 2017 [33] | ICR mice/Male | tMCAO (60 min) | Fetal mouse NSC | 4 d | ipsi-striatum | (100-150)×10^5^ | Unknown | 1 | mNSS; infarct volume |
| Ma 2015 [34] * | C57BL/6 mice/Male | Photothrombotic ischemia stroke | Neonatal mouse NSC | 2 d | contra-hippocampus | ＞400×10^5^ | Unknown | 1 | Rotarod test; infarct volume |
| Mine 2013 [35] * | Nude rats/Male | tMCAO (60 min) | Human fetal NSPC | 2 d | ipsi-striatum | (6.7-8)×10^5^ | No | 1 | Cylinder test |
| Mochizuki 2008 [36] | Wistar rats/ Male | Microsphere-induced permanent occlusion | Fetal rat NPC | 10 min | ipsi-hippocampus | (3.8-4.5)×10^5^ | Unknown | 1 | mNSS; infarct volume |
| Mochizuki 2011 [37] | Wistar rats/ Male | Microsphere-induced permanent occlusion | Fetal rat NPC | 7 d | ipsi-hippocampus | (3.8-4.5)×10^5^ | Unknown | 1 | mNSS; infarct volume |
| Mohamad 2013 [38] * | C57BL/6 mice | Unilateral pMCAO and bilateral CCAO (7 min) | Human iPSC-NPC | 7 d | ipsi-cortex | 160×10^5^ | No | 1 |  |
| Muneton-Gomez 2012 [39] * | Wistar rats/ Male | ET-1 induced transient occlusion | Fetal mouse NSC | 2 hrs | ipsi-striatum | (2.8-4)×10^5^ | Unknown | 1 | Cylinder test |
| Nakagomi 2009 [40] | SCID mice/Male | pMCAO | Adult mouse NSC | 7 d | ipsi-cortex | ＜5×10^5^ | No | 1 |  |
| Oki 2012 [41] * | C57BL/6 mice | tMCAO (30 min) | Human iPSC-NSC | 7 d | ipsi-striatum | (40-50)×10^5^ | Yes | 1 | Infarct volume |
| Polentes 2012 [42] | SD rats/Male | tMCAO (90 min) | Human iPSC-NPC | 7 d | ipsi-striatum | 3.6×10^5^ | Yes | 2 | Infarct volume |
|  |  |  | Human ESC-NPC |  |  |  |  |  |  |
| Sakata 2012 [43] | C57BL/6 mice/Male | tMCAO (45 min) | Embryonic mouse NSC | 6 hrs | ipsi-cortex | (120-150)×10^5^ | Unknown | 1 | Rotarod test; mNSS; infarct volume |
| Sakata 2012 [44] * | SD rats/Male | tMCAO (90 min) | Fetal rat NPC | 6 hrs | ipsi-cortex | (3.6-3.8)×10^5^ | Unknown | 1 | Rotarod test; infarct volume |
| Somaa 2017 [45] * | Nude rat | ET-1 induced transient occlusion | Human ESC-NPC | 3 wks | ipsi-cortex | (3.3-4)×10^5^ | Unknown | 1 | Rotarod test; cylinder test |
| Tajiri 2014 [46] | SD rats/Male | tMCAO (60 min) | Human fetal spinal cord NSC | 7 d | ipsi-striatum | (13-26)×10^5^ | Yes | 3 |  |
|  |  |  |  |  |  | (26-50)×10^5^ |  |  |  |
|  |  |  |  |  |  | ＞50×10^5^ |  |  |  |
| Takahashi 2008 [47] | Wistar rats/ Male | tMCAO (90 min) | Rat adult NPC | 1 hr | ipsi-cortex | (3.6-4)×10^5^ | No | 2 | Rotarod test; cylinder test; infarct volume |
|  |  |  | Rat embryonic NPC |  |  |  |  |  |  |
| Tang 2014 [48] | SD rats/Male | tMCAO (120 min) | Embryonic mouse NPC | 24 hrs | ipsi-striatum | 36×10^5^ | Yes | 2 | Rotarod test; mNSS; infarct volume |
| Tatarishvili 2014 [49] * | SD rats/Male | Distal pMCAO and bilateral CCAO (30 min) | Human iPSC-NSC | 2 d | ipsi-cortex | (5-9.4)×10^5^ | Yes | 1 | Cylinder test; infarct volume |
| Theus 2008 [50] * | Wistar rats/Male | tMCAO (120 min) | Mouse ESC-NSC | 2 d | ipsi-cortex and striatum | (14-14.5)×10^5^ | Yes | 1 | Rotarod test; mNSS |
| Yamane 2011 [51] | Mongolian gerbil/Male | Unilateral CCAO for 10 min | Human fetal NSPC | 4 d | peri-infarct | (6.9-8.3)×10^5^ | Yes | 1 |  |
| Yang 2009 [52] * | ICR mice/Male | Bilateral CCAO for 20 min | Mouse ESC-NSC | 3 d | ipsi- caudate putamen | (167-200)×10^5^ | Unknown | 1 |  |
| Yao 2015 [53] * | SD rats/Male | pMCAO | Embryonic mouse NSC | 2 d | ipsi-cortex | (33-37)×10^5^ | Unknown | 1 | mNSS |
| Yuan 2013 [54] * | SD rats/Male | tMCAO (120 min) | Human iPSC-NSC | ＜1 hr | ipsi-striatum | (33-37)×10^5^ | Yes | 1 |  |
| Zhang 2017 [55] * | SD rats | tMCAO (120 min) | Fetal rat NSC | 2 d | contra-striatum | (17.9-20)×10^5^ | No | 1 | mNSS; infarct volume |
| Zhang 2018 [56] * | SD rats/Male | tMCAO (90 min) | Fetal rat NSC | 3 d | ipsi-striatum | (28.6-33.3)×10^5^ | No | 1 | mNSS; rotarod test; infarct volume |
| Zhang 2009 [57] | SD rats/Male | pMCAO | Human fetal NSC | 24 hrs | ipsi-cortex | (1.4-2)×10^5^ | Unknown | 1 | mNSS; infarct volume |
| Zhang 2017 [58] | ICR mice/Male | tMCAO (120 min) | Embryonic mouse NSC | 24 hrs | ipsi-striatum | (333-400)×10^5^ | No | 1 | mNSS; rotarod test; infarct volume |
| Zhang 2008 [59] | SD rats/Male | tMCAO (120 min) | Embryonic rat NSC | 7 d | ipsi-striatum | (8-8.7)×10^5^ | Unknown | 1 | mNSS |
| Zhao 2010 [60] | Wistar rats/ Female | tMCAO (120 min) | Embryonic rat NSC | 2 d | ipsi-hippocampus | 2×10^5^ | Unknown | 1 | mNSS; infarct volume |
| Zhu 2011 [61] | SD rats/Male | tMCAO (120 min) | Embryonic rat NSC | 3 d | ipsi-striatum | (33-40)×10^5^ | No | 1 | mNSS |
| Zhu 2005 [62] * | SD rats/Male | tMCAO (120 min) | Embryonic rat NSC | 3 d | ipsi-cortex | (6.7-8)×10^5^ | No | 1 | mNSS |

These documents were ranked in alphabetical order of the authors. *(t/p)MCAO (transient/permanent)* middle cerebral artery occlusion*, CCAO* common carotid artery occlusion, *NPC* neural progenitor cell or neural precursor, *NSC* neural stem cell, *NSPC* neural stem/progenitor cells, *iPSC* induced pluripotent stem cell, *ESC* embryonic stem cell, *d* days, *hr(s)* hour(s), *wks* weeks, *ipsi* ipsilateral, *contra* contralateral, *mNSS* modified neurological severity score.

An asterisk symbol (*) companied with the author name in the item of “study ID” meant studies with statement of treatment related serious adverse events.

**Supplementary References**

[1] H.C. Abeysinghe, L. Bokhari, A. Quigley, M. Choolani, J. Chan, G.J. Dusting, J.M. Crook, N.R. Kobayashi, C.L. Roulston, Pre-differentiation of human neural stem cells into GABAergic neurons prior to transplant results in greater repopulation of the damaged brain and accelerates functional recovery after transient ischemic stroke, Stem cell research &amp; therapy, 6 (2015) 186.

[2] R.H. Andres, N. Horie, W. Slikker, H. Keren-Gill, K. Zhan, G. Sun, N.C. Manley, M.P. Pereira, L.A. Sheikh, E.L. McMillan, B.T. Schaar, C.N. Svendsen, T.M. Bliss, G.K. Steinberg, Human neural stem cells enhance structural plasticity and axonal transport in the ischaemic brain, Brain, 134 (2011) 1777-1789.

[3] I.L. Augestad, A.K. Nyman, A.I. Costa, S.C. Barnett, A. Sandvig, A.K. Haberg, I. Sandvig, Effects of Neural Stem Cell and Olfactory Ensheathing Cell Co-transplants on Tissue Remodelling After Transient Focal Cerebral Ischemia in the Adult Rat, Neurochem Res, (2017).

[4] Q. Cai, Z. Chen, P. Song, L. Wu, L. Wang, G. Deng, B. Liu, Q. Chen, Co-transplantation of hippocampal neural stem cells and astrocytes and microvascular endothelial cells improve the memory in ischemic stroke rat, Int. J. Clin. Exp. Med., 8 (2015) 13109-13117.

[5] D.J. Chang, N. Lee, I.H. Park, C. Choi, I. Jeon, J. Kwon, S.H. Oh, D.A. Shin, J.T. Do, D.R. Lee, H. Lee, H. Moon, K.S. Hong, G.Q. Daley, J. Song, Therapeutic potential of human induced pluripotent stem cells in experimental stroke, Cell Transplant, 22 (2013) 1427-1440.

[6] D.-J. Chang, S.-H. Oh, N. Lee, C. Choi, I. Jeon, H.S. Kim, D.A. Shin, S.E. Lee, D. Kim, J. Song, Contralaterally transplanted human embryonic stem cell-derived neural precursor cells (ENStem-A) migrate and improve brain functions in stroke-damaged rats, Exp. Mol. Med., 45 (2013).

[7] M.J. Chau, T.C. Deveau, M. Song, X. Gu, D. Chen, L. Wei, iPSC Transplantation increases regeneration and functional recovery after ischemic stroke in neonatal rats, Stem Cells, 32 (2014) 3075-3087.

[8] M. Chau, T.C. Deveau, M.K. Song, Z.Z. Wei, X.H. Gu, S.P. Yu, L. Wei, Transplantation of iPS cell-derived neural progenitors overexpressing SDF-1 alpha increases regeneration and functional recovery after ischemic stroke, Oncotarget, 8 (2017) 97537-97553.

[9] L. Chen, R. Qiu, L. Li, D. He, H. Lv, X. Wu, N. Gu, The role of exogenous neural stem cells transplantation in cerebral ischemic stroke, J Biomed Nanotechnol, 10 (2014) 3219-3230.

[10] M.M. Daadi, J.Q. Klausner, B. Bajar, I. Goshen, C. Lee-Messer, S.Y. Lee, M.C. Winge, C. Ramakrishnan, M. Lo, G. Sun, K. Deisseroth, G.K. Steinberg, Optogenetic Stimulation of Neural Grafts Enhances Neurotransmission and Downregulates the Inflammatory Response in Experimental Stroke Model, Cell Transplant, 25 (2016) 1371-1380.

[11] M.M. Daadi, S.H. Lee, A. Arac, B.A. Grueter, R. Bhatnagar, A.L. Maag, B. Schaar, R.C. Malenka, T.D. Palmer, G.K. Steinberg, Functional engraftment of the medial ganglionic eminence cells in experimental stroke model, Cell Transplant, 18 (2009) 815-826.

[12] M.M. Daadi, A.L. Maag, G.K. Steinberg, Adherent self-renewable human embryonic stem cell-derived neural stem cell line: functional engraftment in experimental stroke model, PLoS One, 3 (2008) e1644.

[13] T.R. Doeppner, M. El Aanbouri, G.P. Dietz, J. Weise, S. Schwarting, M. Bahr, Transplantation of TAT-Bcl-xL-transduced neural precursor cells: long-term neuroprotection after stroke, Neurobiol Dis, 40 (2010) 265-276.

[14] T.R. Doeppner, T.A. Ewert, L. Tonges, J. Herz, A. Zechariah, A. ElAli, A.K. Ludwig, B. Giebel, F. Nagel, G.P. Dietz, J. Weise, D.M. Hermann, M. Bahr, Transduction of neural precursor cells with TAT-heat shock protein 70 chaperone: therapeutic potential against ischemic stroke after intrastriatal and systemic transplantation, Stem Cells, 30 (2012) 1297-1310.

[15] T.R. Doeppner, B. Kaltwasser, M.K. Teli, E.H. Sanchez-Mendoza, E. Kilic, M. Bahr, D.M. Hermann, Post-stroke transplantation of adult subventricular zone derived neural progenitor cells--A comprehensive analysis of cell delivery routes and their underlying mechanisms, Exp Neurol, 273 (2015) 45-56.

[16] T.R. Doeppner, M. Doehring, B. Kaltwasser, A. Majid, F. Lin, M. Bahr, E. Kilic, D.M. Hermann, Ischemic Post-Conditioning Induces Post-Stroke Neuroprotection via Hsp70-Mediated Proteasome Inhibition and Facilitates Neural Progenitor Cell Transplantation, Molecular Neurobiology, 54 (2017) 6061-6073.

[17] D. Drury-Stewart, M. Song, O. Mohamad, Y. Guo, X. Gu, D. Chen, L. Wei, Highly efficient differentiation of neural precursors from human embryonic stem cells and benefits of transplantation after ischemic stroke in mice, Stem cell research &amp; therapy, 4 (2013) 93.

[18] A. Eckert, L. Huang, R. Gonzalez, H.-S. Kim, M.H. Hamblin, J.-P. Lee, Bystander Effect Fuels Human Induced Pluripotent Stem Cell-Derived Neural Stem Cells to Quickly Attenuate Early Stage Neurological Deficits After Stroke, Stem cells translational medicine, 4 (2015) 841-851.

[19] M. Fujimoto, H. Hayashi, Y. Takagi, M. Hayase, T. Marumo, M. Gomi, M. Nishimura, H. Kataoka, J. Takahashi, N. Hashimoto, K. Nozaki, S. Miyamoto, Transplantation of telencephalic neural progenitors induced from embryonic stem cells into subacute phase of focal cerebral ischemia, Laboratory investigation; a journal of technical methods and pathology, 92 (2012) 522-531.

[20] M. Gomi, Y. Takagi, A. Morizane, D. Doi, M. Nishimura, S. Miyamoto, J. Takahashi, Functional recovery of the murine brain ischemia model using human induced pluripotent stem cell-derived telencephalic progenitors, Brain Res, 1459 (2012) 52-60.

[21] Y. Guan, H. Zou, X. Chen, C. Zhao, J. Wang, Y. Cai, P. Chan, L. Chen, Y.A. Zhang, Ischemia, Immunosuppression, and SSEA-1-Negative Cells All Contribute to Tumors Resulting From Mouse Embryonic Stem Cell-Derived Neural Progenitor Transplantation, J. Neurosci. Res., 92 (2014) 74-85.

[22] Y. Hermanto, T. Sunohara, A. Faried, Y. Takagi, J. Takahashi, T. Maki, S. Miyamoto, Transplantation of feeder-free human induced pluripotent stem cell-derived cortical neuron progenitors in adult male Wistar rats with focal brain ischemia, Journal of neuroscience research, 96 (2018) 863-874.

[23] A.U. Hicks, R.S. Lappalainen, S. Narkilahti, R. Suuronen, D. Corbett, J. Sivenius, O. Hovatta, J. Jolkkonen, Transplantation of human embryonic stem cell-derived neural precursor cells and enriched environment after cortical stroke in rats: cell survival and functional recovery, Eur. J. Neurosci., 29 (2009) 562-574.

[24] A.U. Hicks, C.L. MacLellan, G.A. Chernenko, D. Corbett, Long-term assessment of enriched housing and subventricular zone derived cell transplantation after focal ischemia in rats, Brain Res., 1231 (2008) 103-112.

[25] B. Hou, J. Ma, X. Guo, F. Ju, J. Gao, D. Wang, J. Liu, X. Li, S. Zhang, H. Ren, Exogenous Neural Stem Cells Transplantation as a Potential Therapy for Photothrombotic Ischemia Stroke in Kunming Mice Model, Mol. Neurobiol., (2016) 1-9.

[26] L. Huang, S. Wong, E.Y. Snyder, M.H. Hamblin, J.P. Lee, Human neural stem cells rapidly ameliorate symptomatic inflammation in early-stage ischemic-reperfusion cerebral injury, Stem cell research &amp; therapy, 5 (2014) 129.

[27] S. Ishibashi, M. Sakaguchi, T. Kuroiwa, M. Yamasaki, Y. Kanemura, I. Shizuko, T. Shimazaki, M. Onodera, H. Okano, H. Mizusawa, Human neural stem/progenitor cells, expanded in long-term neurosphere culture, promote functional recovery after focal ischemia in Mongolian gerbils, J Neurosci Res, 78 (2004) 215-223.

[28] M.B. Jensen, H. Yan, R. Krishnaney-Davison, A. Al Sawaf, S.-C. Zhang, Survival and Differentiation of Transplanted Neural Stem Cells Derived from Human Induced Pluripotent Stem Cells in A Rat Stroke Model, Journal of Stroke & Cerebrovascular Diseases, 22 (2013) 304-308.

[29] M. Kameda, T. Shingo, K. Takahashi, K. Muraoka, K. Kurozumi, T. Yasuhara, T. Maruo, T. Tsuboi, T. Uozumi, T. Matsui, Y. Miyoshi, H. Hamada, I. Date, Adult neural stem and progenitor cells modified to secrete GDNF can protect, migrate and integrate after intracerebral transplantation in rats with transient forebrain ischemia, The European journal of neuroscience, 26 (2007) 1462-1478.

[30] D.Y. Kim, S.H. Park, S.U. Lee, D.H. Choi, H.W. Park, S.H. Paek, H.Y. Shin, E.Y. Kim, S.P. Park, J.H. Lim, Effect of human embryonic stem cell-derived neuronal precursor cell transplantation into the cerebral infarct model of rat with exercise, Neurosci. Res., 58 (2007) 164-175.

[31] H.-S. Kim, S.-M. Choi, W. Yang, D.-S. Kim, D.R. Lee, S.-R. Cho, D.-W. Kim, PSA-NCAM(+) Neural Precursor Cells from Human Embryonic Stem Cells Promote Neural Tissue Integrity and Behavioral Performance in A Rat Stroke Model, Stem Cell Rev. Rep., 10 (2014) 761-771.

[32] V.W. Lau, S.R. Platt, H.E. Grace, E.W. Baker, F.D. West, Human iNPC therapy leads to improvement in functional neurologic outcomes in a pig ischemic stroke model, Brain and Behavior, 8 (2018).

[33] Y. Lu, L. Jiang, W. Li, M. Qu, Y. Song, X. He, Z. Zhang, G.Y. Yang, Y. Wang, Optogenetic Inhibition of Striatal Neuronal Activity Improves the Survival of Transplanted Neural Stem Cells and Neurological Outcomes after Ischemic Stroke in Mice, Stem Cells International, 2017 (2017).

[34] J. Ma, J. Gao, B. Hou, J. Liu, S. Chen, G. Yan, H. Ren, Neural stem cell transplantation promotes behavioral recovery in a photothrombosis stroke model, Int. J. Clin. Exp. Pathol., 8 (2015) 7838-7848.

[35] Y. Mine, J. Tatarishvili, K. Oki, E. Monni, Z. Kokaia, O. Lindvall, Grafted human neural stem cells enhance several steps of endogenous neurogenesis and improve behavioral recovery after middle cerebral artery occlusion in rats, Neurobiol Dis, 52 (2013) 191-203.

[36] N. Mochizuki, N. Takagi, K. Kurokawa, C. Onozato, Y. Moriyama, K. Tanonaka, S. Takeo, Injection of neural progenitor cells improved learning and memory dysfunction after cerebral ischemia, Exp Neurol, 211 (2008) 194-202.

[37] N. Mochizuki, Y. Moriyama, N. Takagi, S. Takeo, K. Tanonaka, Intravenous injection of neural progenitor cells improves cerebral ischemia-induced learning dysfunction, Biological &amp; pharmaceutical bulletin, 34 (2011) 260-265.

[38] O. Mohamad, D. Drury-Stewart, M. Song, B. Faulkner, D. Chen, S.P. Yu, L. Wei, Vector-Free and Transgene-Free Human iPS Cells Differentiate into Functional Neurons and Enhance Functional Recovery after Ischemic Stroke in Mice, PLoS One, 8 (2013).

[39] V.C. Muneton-Gomez, E. Doncel-Perez, A.P. Fernandez, J. Serrano, A. Pozo-Rodrigalvarez, L. Vellosillo-Huerta, J.S. Taylor, G.P. Cardona-Gomez, M. Nieto-Sampedro, R. Martinez-Murillo, Neural differentiation of transplanted neural stem cells in a rat model of striatal lacunar infarction: light and electron microscopic observations, Front Cell Neurosci, 6 (2012) 30.

[40] N. Nakagomi, T. Nakagomi, S. Kubo, A. Nakano-Doi, O. Saino, M. Takata, H. Yoshikawa, D.M. Stern, T. Matsuyama, A. Taguchi, Endothelial cells support survival, proliferation, and neuronal differentiation of transplanted adult ischemia-induced neural stem/progenitor cells after cerebral infarction, Stem Cells, 27 (2009) 2185-2195.

[41] K. Oki, J. Tatarishvili, J. Wood, P. Koch, S. Wattananit, Y. Mine, E. Monni, D. Tornero, H. Ahlenius, J. Ladewig, O. Brustle, O. Lindvall, Z. Kokaia, Human-induced pluripotent stem cells form functional neurons and improve recovery after grafting in stroke-damaged brain, Stem Cells, 30 (2012) 1120-1133.

[42] J. Polentes, P. Jendelova, M. Cailleret, H. Braun, N. Romanyuk, P. Tropel, M. Brenot, V. Itier, C. Seminatore, K. Baldauf, K. Turnovcova, D. Jirak, M. Teletin, J. Come, J. Tournois, K. Reymann, E. Sykova, S. Viville, B. Onteniente, Human induced pluripotent stem cells improve stroke outcome and reduce secondary degeneration in the recipient brain, Cell Transplant, 21 (2012) 2587-2602.

[43] H. Sakata, P. Narasimhan, K. Niizuma, C.M. Maier, T. Wakai, P.H. Chan, Interleukin 6-preconditioned neural stem cells reduce ischaemic injury in stroke mice, Brain, 135 (2012) 3298-3310.

[44] H. Sakata, K. Niizuma, H. Yoshioka, G.S. Kim, J.E. Jung, M. Katsu, P. Narasimhan, C.M. Maier, Y. Nishiyama, P.H. Chan, Minocycline-preconditioned neural stem cells enhance neuroprotection after ischemic stroke in rats, The Journal of neuroscience : the official journal of the Society for Neuroscience, 32 (2012) 3462-3473.

[45] F.A. Somaa, T.Y. Wang, J.C. Niclis, K.F. Bruggeman, J.A. Kauhausen, H. Guo, S. McDougall, R.J. Williams, D.R. Nisbet, L.H. Thompson, C.L. Parish, Peptide-Based Scaffolds Support Human Cortical Progenitor Graft Integration to Reduce Atrophy and Promote Functional Repair in a Model of Stroke, Cell reports, 20 (2017) 1964-1977.

[46] N. Tajiri, D.M. Quach, Y. Kaneko, S. Wu, D. Lee, T. Lam, K.L. Hayama, T.G. Hazel, K. Johe, M.C. Wu, C.V. Borlongan, Behavioral and histopathological assessment of adult ischemic rat brains after intracerebral transplantation of NSI-566RSC cell lines, PLoS One, 9 (2014) e91408.

[47] K. Takahashi, T. Yasuhara, T. Shingo, K. Muraoka, M. Kameda, A. Takeuchi, A. Yano, K. Kurozumi, T. Agari, Y. Miyoshi, K. Kinugasa, I. Date, Embryonic neural stem cells transplanted in middle cerebral artery occlusion model of rats demonstrated potent therapeutic effects, compared to adult neural stem cells, Brain Res, 1234 (2008) 172-182.

[48] Y. Tang, J. Wang, X. Lin, L. Wang, B. Shao, K. Jin, Y. Wang, G.Y. Yang, Neural stem cell protects aged rat brain from ischemia-reperfusion injury through neurogenesis and angiogenesis, Journal of cerebral blood flow and metabolism : official journal of the International Society of Cerebral Blood Flow and Metabolism, 34 (2014) 1138-1147.

[49] J. Tatarishvili, K. Oki, E. Monni, P. Koch, T. Memanishvili, A.-M. Buga, V. Verma, A. Popa-Wagner, O. Bruestle, O. Lindvall, Z. Kokaia, Human induced pluripotent stem cells improve recovery in stroke-injured aged rats, Restor. Neurol. Neurosci., 32 (2014) 547-558.

[50] M.H. Theus, L. Wei, L. Cui, K. Francis, X. Hu, C. Keogh, S.P. Yu, In vitro hypoxic preconditioning of embryonic stem cells as a strategy of promoting cell survival and functional benefits after transplantation into the ischemic rat brain, Exp Neurol, 210 (2008) 656-670.

[51] J. Yamane, S. Ishibashi, M. Sakaguchi, T. Kuroiwa, Y. Kanemura, M. Nakamura, H. Miyoshi, K. Sawamoto, Y. Toyama, H. Mizusawa, H. Okano, Transplantation of human neural stem/progenitor cells overexpressing galectin-1 improves functional recovery from focal brain ischemia in the Mongolian gerbil, Mol Brain, 4 (2011) 35.

[52] T. Yang, K.S. Tsang, W.S. Poon, H.K. Ng, Neurotrophism of bone marrow stromal cells to embryonic stem cells: noncontact induction and transplantation to a mouse ischemic stroke model, Cell Transplant, 18 (2009) 391-404.

[53] H. Yao, M. Gao, J.H. Ma, M.Y. Zhang, S.W. Li, B.S. Wu, X.H. Nie, J. Jiao, H. Zhao, S.S. Wang, Y.Y. Yang, Y.S. Zhang, Y.L. Sun, M.S. Wicha, A.E. Chang, S.R. Gao, Q. Li, R.X. Xu, Transdifferentiation-Induced Neural Stem Cells Promote Recovery of Middle Cerebral Artery Stroke Rats, PLoS One, 10 (2015).

[54] T. Yuan, W. Liao, N.-H. Feng, Y.-L. Lou, X. Niu, A.-J. Zhang, Y. Wang, Z.-F. Deng, Human induced pluripotent stem cell-derived neural stem cells survive, migrate, differentiate, and improve neurologic function in a rat model of middle cerebral artery occlusion, Stem Cell Res. Ther., 4 (2013).

[55] F. Zhang, X.H. Duan, L.J. Lu, X. Zhang, M.W. Chen, J.J. Mao, M.H. Cao, J. Shen, In Vivo Long-Term Tracking of Neural Stem Cells Transplanted into an Acute Ischemic Stroke model with Reporter Gene-Based Bimodal MR and Optical Imaging, Cell Transplantation, 26 (2017) 1648-1662.

[56] G.L. Zhang, X.Y. Guo, L.K. Chen, B.Q. Li, B. Gu, H. Wang, G.J. Wu, J. Kong, W.H. Chen, Y.B. Yu, Interferon- Promotes Neuronal Repair by Transplanted Neural Stem Cells in Ischemic Rats, Stem Cells and Development, 27 (2018) 355-366.

[57] P. Zhang, J. Li, Y. Liu, X. Chen, Q. Kang, J. Zhao, W. Li, Human neural stem cell transplantation attenuates apoptosis and improves neurological functions after cerebral ischemia in rats, Acta Anaesthesiol Scand, 53 (2009) 1184-1191.

[58] T. Zhang, X. Yang, T. Liu, J. Shao, N. Fu, A. Yan, K. Geng, W. Xia, Adjudin-preconditioned neural stem cells enhance neuroprotection after ischemia reperfusion in mice, Stem cell research & therapy, 8 (2017) 248.

[59] Z.-h. Zhang, R.-z. Wang, R.-z. Wang, G.-l. Li, J.-j. Wei, Z.-j. Li, M. Feng, J. Kang, W.-c. Du, W.-b. Ma, Y.-n. Li, Y. Yang, Y.-g. Kong, Transplantation of neural stem cells modified by human neurotrophin-3 promotes functional recovery after transient focal cerebral ischemia in rats, Neurosci. Lett., 444 (2008) 227-230.

[60] Y. Zhao, S.T. Yao, S.J. Wang, Neural stem cell transplantation in the hippocampus of rats with cerebral ischemia/reperfusion injury Activation of the phosphatidylinositol-3 kinase/Akt pathway and increased brain-derived neurotrophic factor expression, Neural Regen. Res., 5 (2010) 1605-1610.

[61] J.M. Zhu, Y.Y. Zhao, S.D. Chen, W.H. Zhang, L. Lou, X. Jin, Functional Recovery after Transplantation of Neural Stem Cells Modified by Brain-derived Neurotrophic Factor in Rats with Cerebral Ischaemia, J. Int. Med. Res., 39 (2011) 488-498.

[62] W. Zhu, Y. Mao, Y. Zhao, L.F. Zhou, Y. Wang, J.H. Zhu, Y. Zhu, G.Y. Yang, Transplantation of vascular endothelial growth factor-transfected neural stem cells into the rat brain provides neuroprotection after transient focal cerebral ischemia, Neurosurgery, 57 (2005) 325-333; discussion 325-333.
