# Supplementary material for: Intraparenchymal Neural Stem/Progenitor Cell Transplantation for Ischemic Stroke Animals: A Meta-Analysis and Systematic Review
Source: Stem Cells Int. 2018 Oct 2;2018:4826407. doi: 10.1155/2018/4826407 (PMC6189667; doi:10.1155/2018/4826407)
Supplement: Supplementary 5 — Table S4: study quality assessment referred to the CAMARADES checklists. [file 4826407.f5.docx]

Table S4. Study Quality assessment referred to the CAMARADES checklists

| Study ID | Publication on a peer-reviewed journal | Control of temperature | Randomization to treatment group | Allocation concealment | Blinded assessment of outcome | Avoidance neuroprotective anesthetics | Use of animal with relevant comorbidities | Sample size calculation | Compliance with animal welfare regulations | Statement of conflict of interest | **Score** |
| --- | --- | --- | --- | --- | --- | --- | --- | --- | --- | --- | --- |
| Abeysinghe 2015 [1] | √ | √ | √ |  | √ |  |  |  | √ | √ | **6** |
| Andres 2011 [2] | √ | √ | √ |  | √ | √ |  |  | √ |  | 6 |
| Augestad 2017 [3] | √ | √ | √ | √ | √ | √ |  |  | √ | √ | 8 |
| Cai 2015 [4] | √ | √ | √ |  |  |  |  |  | √ | √ | 5 |
| Chang 2013 [5] | √ | √ |  |  |  |  |  |  | √ | √ | 4 |
| Chang 2013 [6] | √ | √ |  |  |  |  |  |  | √ | √ | 4 |
| Chau 2014 [7] | √ |  |  |  |  | √ |  |  | √ | √ | 4 |
| Chau 2017 [8] | √ |  |  |  |  | √ |  |  | √ | √ | 4 |
| Chen 2014 [9] | √ |  | √ |  |  | √ |  |  | √ |  | 4 |
| Daadi 2016 [10] | √ | √ |  |  | √ |  |  |  | √ | √ | 5 |
| Daadi 2009 [11] | √ | √ |  |  | √ |  |  |  | √ |  | 4 |
| Daadi 2008 [12] | √ | √ |  |  | √ |  |  |  | √ | √ | 5 |
| Doeppner 2010 [13] | √ | √ |  |  | √ |  |  |  | √ |  | 4 |
| Doeppner 2012 [14] | √ | √ |  |  | √ |  |  |  | √ | √ | 5 |
| Doeppner 2015 [15] | √ | √ | √ | √ | √ |  |  |  | √ |  | 6 |
| Doeppner 2017 [16] | √ | √ |  |  | √ |  |  |  | √ | √ | 5 |
| Drury-Stewart 2013 [17] | √ | √ |  |  | √ | √ |  |  | √ | √ | 6 |
| Eckert 2015 [18] | √ | √ |  |  |  | √ |  |  | √ | √ | 5 |
| Fujimoto 2012 [19] | √ | √ |  |  |  | √ |  |  | √ | √ | 5 |
| Gomi 2012 [20] | √ | √ |  |  |  | √ |  |  | √ |  | 4 |
| Guan 2014 [21] | √ | √ |  |  | √ | √ |  |  | √ |  | 5 |
| Hermanto 2017 [22] | √ | √ | √ |  | √ | √ |  |  | √ | √ | 7 |
| Hicks 2009 [23] | √ | √ |  |  |  | √ |  |  | √ |  | 4 |
| Hicks 2008 [24] | √ | √ | √ |  | √ | √ |  |  | √ |  | 6 |
| Hou 2016 [25] | √ | √ |  |  |  |  |  |  | √ | √ | 4 |
| Huang 2014 [26] | √ | √ |  |  |  | √ |  |  | √ | √ | 5 |
| Ishibashi 2004 [27] | √ |  | √ |  | √ | √ |  |  | √ |  | 5 |
| Jensen 2013 [28] | √ | √ | √ |  | √ | √ |  |  | √ |  | 6 |
| Kameda 2007 [29] | √ |  |  |  |  | √ |  |  | √ |  | 3 |
| Kim 2007 [30] | √ | √ | √ |  | √ |  |  |  | √ |  | 5 |
| Kim 2014 [31] | √ |  | √ |  |  | √ |  |  | √ | √ | 5 |
| Lau 2018 [32] | √ | √ |  |  | √ | √ |  |  | √ |  | 5 |
| Lu 2017 [33] | √ | √ | √ |  |  | √ |  |  | √ | √ | 6 |
| Ma 2015 [34] | √ | √ |  |  | √ |  |  |  | √ | √ | 5 |
| Mine 2013 [35] | √ | √ |  |  | √ | √ |  |  | √ |  | 5 |
| Mochizuki 2008 [36] | √ |  |  |  | √ | √ |  |  | √ |  | 4 |
| Mochizuki 2011 [37] | √ |  |  |  | √ |  |  |  | √ |  | 3 |
| Mohamad 2013 [38] | √ | √ |  |  | √ | √ |  |  | √ | √ | 6 |
| Muneton-Gomez 2012 [39] | √ |  |  |  | √ | √ |  |  | √ | √ | 5 |
| Nakagomi 2009 [40] | √ | √ |  |  | √ | √ |  |  | √ | √ | 6 |
| Oki 2012 [41] | √ | √ |  |  |  | √ |  |  | √ | √ | 5 |
| Polentes 2012 [42] | √ | √ | √ |  | √ | √ |  |  | √ | √ | 7 |
| Sakata 2012 [43] | √ | √ | √ |  | √ | √ |  |  | √ |  | 6 |
| Sakata 2012 [44] | √ | √ |  |  | √ | √ |  |  | √ | √ | 6 |
| Somaa 2017 [45] | √ |  |  |  |  |  |  |  | √ |  | 2 |
| Tajiri 2014 [46] | √ | √ | √ |  | √ | √ |  |  | √ | √ | 7 |
| Takahashi 2008 [47] | √ | √ |  |  |  | √ |  |  | √ |  | 4 |
| Tang 2014 [48] | √ | √ | √ |  | √ |  |  |  | √ | √ | 6 |
| Tatarishvili 2014 [49] | √ | √ |  |  | √ | √ |  |  | √ |  | 5 |
| Theus 2008 [50] | √ | √ |  |  |  | √ |  |  | √ |  | 4 |
| Yamane 2011 [51] | √ |  | √ |  | √ | √ |  |  | √ | √ | 6 |
| Yang 2009 [52] | √ |  | √ |  | √ |  |  |  | √ |  | 4 |
| Yao 2015 [53] | √ | √ |  |  | √ |  |  |  | √ | √ | 5 |
| Yuan 2013 [54] | √ | √ | √ |  |  |  |  |  | √ | √ | 5 |
| Zhang 2017 [55] | √ |  | √ |  | √ |  |  |  | √ | √ | 5 |
| Zhang 2018 [56] | √ | √ | √ |  | √ |  |  |  | √ | √ | 6 |
| Zhang 2009 [57] | √ | √ | √ |  | √ | √ |  |  | √ |  | 6 |
| Zhang 2017 [58] | √ | √ | √ |  | √ |  |  |  | √ | √ | 6 |
| Zhang 2008 [59] | √ | √ | √ | √ | √ | √ |  |  | √ |  | 7 |
| Zhao 2010 [60] | √ | √ | √ |  |  |  |  |  | √ |  | 4 |
| Zhu 2011 [61] | √ | √ | √ |  |  | √ |  |  | √ | √ | 6 |
| Zhu 2005 [62] | √ | √ |  |  |  | √ |  |  | √ |  | 4 |
| **Total** | 62 | 49 | 27 | 3 | 39 | 38 | 0 | 0 | 62 | 35 | 316 |
| **Percentage** | 100 | 79.0 | 43.5 | 4.8 | 62.9 | 61.3 | 0 | 0 | 100 | 56.5 |  |

CAMARADES: Collaborative Approach to Meta-Analysis and Review of Animal Data from Experimental Studies

**Supplementary References**

[1] H.C. Abeysinghe, L. Bokhari, A. Quigley, M. Choolani, J. Chan, G.J. Dusting, J.M. Crook, N.R. Kobayashi, C.L. Roulston, Pre-differentiation of human neural stem cells into GABAergic neurons prior to transplant results in greater repopulation of the damaged brain and accelerates functional recovery after transient ischemic stroke, Stem cell research &amp; therapy, 6 (2015) 186.

[2] R.H. Andres, N. Horie, W. Slikker, H. Keren-Gill, K. Zhan, G. Sun, N.C. Manley, M.P. Pereira, L.A. Sheikh, E.L. McMillan, B.T. Schaar, C.N. Svendsen, T.M. Bliss, G.K. Steinberg, Human neural stem cells enhance structural plasticity and axonal transport in the ischaemic brain, Brain, 134 (2011) 1777-1789.

[3] I.L. Augestad, A.K. Nyman, A.I. Costa, S.C. Barnett, A. Sandvig, A.K. Haberg, I. Sandvig, Effects of Neural Stem Cell and Olfactory Ensheathing Cell Co-transplants on Tissue Remodelling After Transient Focal Cerebral Ischemia in the Adult Rat, Neurochem Res, (2017).

[4] Q. Cai, Z. Chen, P. Song, L. Wu, L. Wang, G. Deng, B. Liu, Q. Chen, Co-transplantation of hippocampal neural stem cells and astrocytes and microvascular endothelial cells improve the memory in ischemic stroke rat, Int. J. Clin. Exp. Med., 8 (2015) 13109-13117.

[5] D.J. Chang, N. Lee, I.H. Park, C. Choi, I. Jeon, J. Kwon, S.H. Oh, D.A. Shin, J.T. Do, D.R. Lee, H. Lee, H. Moon, K.S. Hong, G.Q. Daley, J. Song, Therapeutic potential of human induced pluripotent stem cells in experimental stroke, Cell Transplant, 22 (2013) 1427-1440.

[6] D.-J. Chang, S.-H. Oh, N. Lee, C. Choi, I. Jeon, H.S. Kim, D.A. Shin, S.E. Lee, D. Kim, J. Song, Contralaterally transplanted human embryonic stem cell-derived neural precursor cells (ENStem-A) migrate and improve brain functions in stroke-damaged rats, Exp. Mol. Med., 45 (2013).

[7] M.J. Chau, T.C. Deveau, M. Song, X. Gu, D. Chen, L. Wei, iPSC Transplantation increases regeneration and functional recovery after ischemic stroke in neonatal rats, Stem Cells, 32 (2014) 3075-3087.

[8] M. Chau, T.C. Deveau, M.K. Song, Z.Z. Wei, X.H. Gu, S.P. Yu, L. Wei, Transplantation of iPS cell-derived neural progenitors overexpressing SDF-1 alpha increases regeneration and functional recovery after ischemic stroke, Oncotarget, 8 (2017) 97537-97553.

[9] L. Chen, R. Qiu, L. Li, D. He, H. Lv, X. Wu, N. Gu, The role of exogenous neural stem cells transplantation in cerebral ischemic stroke, J Biomed Nanotechnol, 10 (2014) 3219-3230.

[10] M.M. Daadi, J.Q. Klausner, B. Bajar, I. Goshen, C. Lee-Messer, S.Y. Lee, M.C. Winge, C. Ramakrishnan, M. Lo, G. Sun, K. Deisseroth, G.K. Steinberg, Optogenetic Stimulation of Neural Grafts Enhances Neurotransmission and Downregulates the Inflammatory Response in Experimental Stroke Model, Cell Transplant, 25 (2016) 1371-1380.

[11] M.M. Daadi, S.H. Lee, A. Arac, B.A. Grueter, R. Bhatnagar, A.L. Maag, B. Schaar, R.C. Malenka, T.D. Palmer, G.K. Steinberg, Functional engraftment of the medial ganglionic eminence cells in experimental stroke model, Cell Transplant, 18 (2009) 815-826.

[12] M.M. Daadi, A.L. Maag, G.K. Steinberg, Adherent self-renewable human embryonic stem cell-derived neural stem cell line: functional engraftment in experimental stroke model, PLoS One, 3 (2008) e1644.

[13] T.R. Doeppner, M. El Aanbouri, G.P. Dietz, J. Weise, S. Schwarting, M. Bahr, Transplantation of TAT-Bcl-xL-transduced neural precursor cells: long-term neuroprotection after stroke, Neurobiol Dis, 40 (2010) 265-276.

[14] T.R. Doeppner, T.A. Ewert, L. Tonges, J. Herz, A. Zechariah, A. ElAli, A.K. Ludwig, B. Giebel, F. Nagel, G.P. Dietz, J. Weise, D.M. Hermann, M. Bahr, Transduction of neural precursor cells with TAT-heat shock protein 70 chaperone: therapeutic potential against ischemic stroke after intrastriatal and systemic transplantation, Stem Cells, 30 (2012) 1297-1310.

[15] T.R. Doeppner, B. Kaltwasser, M.K. Teli, E.H. Sanchez-Mendoza, E. Kilic, M. Bahr, D.M. Hermann, Post-stroke transplantation of adult subventricular zone derived neural progenitor cells--A comprehensive analysis of cell delivery routes and their underlying mechanisms, Exp Neurol, 273 (2015) 45-56.

[16] T.R. Doeppner, M. Doehring, B. Kaltwasser, A. Majid, F. Lin, M. Bahr, E. Kilic, D.M. Hermann, Ischemic Post-Conditioning Induces Post-Stroke Neuroprotection via Hsp70-Mediated Proteasome Inhibition and Facilitates Neural Progenitor Cell Transplantation, Molecular Neurobiology, 54 (2017) 6061-6073.

[17] D. Drury-Stewart, M. Song, O. Mohamad, Y. Guo, X. Gu, D. Chen, L. Wei, Highly efficient differentiation of neural precursors from human embryonic stem cells and benefits of transplantation after ischemic stroke in mice, Stem cell research &amp; therapy, 4 (2013) 93.

[18] A. Eckert, L. Huang, R. Gonzalez, H.-S. Kim, M.H. Hamblin, J.-P. Lee, Bystander Effect Fuels Human Induced Pluripotent Stem Cell-Derived Neural Stem Cells to Quickly Attenuate Early Stage Neurological Deficits After Stroke, Stem cells translational medicine, 4 (2015) 841-851.

[19] M. Fujimoto, H. Hayashi, Y. Takagi, M. Hayase, T. Marumo, M. Gomi, M. Nishimura, H. Kataoka, J. Takahashi, N. Hashimoto, K. Nozaki, S. Miyamoto, Transplantation of telencephalic neural progenitors induced from embryonic stem cells into subacute phase of focal cerebral ischemia, Laboratory investigation; a journal of technical methods and pathology, 92 (2012) 522-531.

[20] M. Gomi, Y. Takagi, A. Morizane, D. Doi, M. Nishimura, S. Miyamoto, J. Takahashi, Functional recovery of the murine brain ischemia model using human induced pluripotent stem cell-derived telencephalic progenitors, Brain Res, 1459 (2012) 52-60.

[21] Y. Guan, H. Zou, X. Chen, C. Zhao, J. Wang, Y. Cai, P. Chan, L. Chen, Y.A. Zhang, Ischemia, Immunosuppression, and SSEA-1-Negative Cells All Contribute to Tumors Resulting From Mouse Embryonic Stem Cell-Derived Neural Progenitor Transplantation, J. Neurosci. Res., 92 (2014) 74-85.

[22] Y. Hermanto, T. Sunohara, A. Faried, Y. Takagi, J. Takahashi, T. Maki, S. Miyamoto, Transplantation of feeder-free human induced pluripotent stem cell-derived cortical neuron progenitors in adult male Wistar rats with focal brain ischemia, Journal of neuroscience research, 96 (2018) 863-874.

[23] A.U. Hicks, R.S. Lappalainen, S. Narkilahti, R. Suuronen, D. Corbett, J. Sivenius, O. Hovatta, J. Jolkkonen, Transplantation of human embryonic stem cell-derived neural precursor cells and enriched environment after cortical stroke in rats: cell survival and functional recovery, Eur. J. Neurosci., 29 (2009) 562-574.

[24] A.U. Hicks, C.L. MacLellan, G.A. Chernenko, D. Corbett, Long-term assessment of enriched housing and subventricular zone derived cell transplantation after focal ischemia in rats, Brain Res., 1231 (2008) 103-112.

[25] B. Hou, J. Ma, X. Guo, F. Ju, J. Gao, D. Wang, J. Liu, X. Li, S. Zhang, H. Ren, Exogenous Neural Stem Cells Transplantation as a Potential Therapy for Photothrombotic Ischemia Stroke in Kunming Mice Model, Mol. Neurobiol., (2016) 1-9.

[26] L. Huang, S. Wong, E.Y. Snyder, M.H. Hamblin, J.P. Lee, Human neural stem cells rapidly ameliorate symptomatic inflammation in early-stage ischemic-reperfusion cerebral injury, Stem cell research &amp; therapy, 5 (2014) 129.

[27] S. Ishibashi, M. Sakaguchi, T. Kuroiwa, M. Yamasaki, Y. Kanemura, I. Shizuko, T. Shimazaki, M. Onodera, H. Okano, H. Mizusawa, Human neural stem/progenitor cells, expanded in long-term neurosphere culture, promote functional recovery after focal ischemia in Mongolian gerbils, J Neurosci Res, 78 (2004) 215-223.

[28] M.B. Jensen, H. Yan, R. Krishnaney-Davison, A. Al Sawaf, S.-C. Zhang, Survival and Differentiation of Transplanted Neural Stem Cells Derived from Human Induced Pluripotent Stem Cells in A Rat Stroke Model, Journal of Stroke & Cerebrovascular Diseases, 22 (2013) 304-308.

[29] M. Kameda, T. Shingo, K. Takahashi, K. Muraoka, K. Kurozumi, T. Yasuhara, T. Maruo, T. Tsuboi, T. Uozumi, T. Matsui, Y. Miyoshi, H. Hamada, I. Date, Adult neural stem and progenitor cells modified to secrete GDNF can protect, migrate and integrate after intracerebral transplantation in rats with transient forebrain ischemia, The European journal of neuroscience, 26 (2007) 1462-1478.

[30] D.Y. Kim, S.H. Park, S.U. Lee, D.H. Choi, H.W. Park, S.H. Paek, H.Y. Shin, E.Y. Kim, S.P. Park, J.H. Lim, Effect of human embryonic stem cell-derived neuronal precursor cell transplantation into the cerebral infarct model of rat with exercise, Neurosci. Res., 58 (2007) 164-175.

[31] H.-S. Kim, S.-M. Choi, W. Yang, D.-S. Kim, D.R. Lee, S.-R. Cho, D.-W. Kim, PSA-NCAM(+) Neural Precursor Cells from Human Embryonic Stem Cells Promote Neural Tissue Integrity and Behavioral Performance in A Rat Stroke Model, Stem Cell Rev. Rep., 10 (2014) 761-771.

[32] V.W. Lau, S.R. Platt, H.E. Grace, E.W. Baker, F.D. West, Human iNPC therapy leads to improvement in functional neurologic outcomes in a pig ischemic stroke model, Brain and Behavior, 8 (2018).

[33] Y. Lu, L. Jiang, W. Li, M. Qu, Y. Song, X. He, Z. Zhang, G.Y. Yang, Y. Wang, Optogenetic Inhibition of Striatal Neuronal Activity Improves the Survival of Transplanted Neural Stem Cells and Neurological Outcomes after Ischemic Stroke in Mice, Stem Cells International, 2017 (2017).

[34] J. Ma, J. Gao, B. Hou, J. Liu, S. Chen, G. Yan, H. Ren, Neural stem cell transplantation promotes behavioral recovery in a photothrombosis stroke model, Int. J. Clin. Exp. Pathol., 8 (2015) 7838-7848.

[35] Y. Mine, J. Tatarishvili, K. Oki, E. Monni, Z. Kokaia, O. Lindvall, Grafted human neural stem cells enhance several steps of endogenous neurogenesis and improve behavioral recovery after middle cerebral artery occlusion in rats, Neurobiol Dis, 52 (2013) 191-203.

[36] N. Mochizuki, N. Takagi, K. Kurokawa, C. Onozato, Y. Moriyama, K. Tanonaka, S. Takeo, Injection of neural progenitor cells improved learning and memory dysfunction after cerebral ischemia, Exp Neurol, 211 (2008) 194-202.

[37] N. Mochizuki, Y. Moriyama, N. Takagi, S. Takeo, K. Tanonaka, Intravenous injection of neural progenitor cells improves cerebral ischemia-induced learning dysfunction, Biological &amp; pharmaceutical bulletin, 34 (2011) 260-265.

[38] O. Mohamad, D. Drury-Stewart, M. Song, B. Faulkner, D. Chen, S.P. Yu, L. Wei, Vector-Free and Transgene-Free Human iPS Cells Differentiate into Functional Neurons and Enhance Functional Recovery after Ischemic Stroke in Mice, PLoS One, 8 (2013).

[39] V.C. Muneton-Gomez, E. Doncel-Perez, A.P. Fernandez, J. Serrano, A. Pozo-Rodrigalvarez, L. Vellosillo-Huerta, J.S. Taylor, G.P. Cardona-Gomez, M. Nieto-Sampedro, R. Martinez-Murillo, Neural differentiation of transplanted neural stem cells in a rat model of striatal lacunar infarction: light and electron microscopic observations, Front Cell Neurosci, 6 (2012) 30.

[40] N. Nakagomi, T. Nakagomi, S. Kubo, A. Nakano-Doi, O. Saino, M. Takata, H. Yoshikawa, D.M. Stern, T. Matsuyama, A. Taguchi, Endothelial cells support survival, proliferation, and neuronal differentiation of transplanted adult ischemia-induced neural stem/progenitor cells after cerebral infarction, Stem Cells, 27 (2009) 2185-2195.

[41] K. Oki, J. Tatarishvili, J. Wood, P. Koch, S. Wattananit, Y. Mine, E. Monni, D. Tornero, H. Ahlenius, J. Ladewig, O. Brustle, O. Lindvall, Z. Kokaia, Human-induced pluripotent stem cells form functional neurons and improve recovery after grafting in stroke-damaged brain, Stem Cells, 30 (2012) 1120-1133.

[42] J. Polentes, P. Jendelova, M. Cailleret, H. Braun, N. Romanyuk, P. Tropel, M. Brenot, V. Itier, C. Seminatore, K. Baldauf, K. Turnovcova, D. Jirak, M. Teletin, J. Come, J. Tournois, K. Reymann, E. Sykova, S. Viville, B. Onteniente, Human induced pluripotent stem cells improve stroke outcome and reduce secondary degeneration in the recipient brain, Cell Transplant, 21 (2012) 2587-2602.

[43] H. Sakata, P. Narasimhan, K. Niizuma, C.M. Maier, T. Wakai, P.H. Chan, Interleukin 6-preconditioned neural stem cells reduce ischaemic injury in stroke mice, Brain, 135 (2012) 3298-3310.

[44] H. Sakata, K. Niizuma, H. Yoshioka, G.S. Kim, J.E. Jung, M. Katsu, P. Narasimhan, C.M. Maier, Y. Nishiyama, P.H. Chan, Minocycline-preconditioned neural stem cells enhance neuroprotection after ischemic stroke in rats, The Journal of neuroscience : the official journal of the Society for Neuroscience, 32 (2012) 3462-3473.

[45] F.A. Somaa, T.Y. Wang, J.C. Niclis, K.F. Bruggeman, J.A. Kauhausen, H. Guo, S. McDougall, R.J. Williams, D.R. Nisbet, L.H. Thompson, C.L. Parish, Peptide-Based Scaffolds Support Human Cortical Progenitor Graft Integration to Reduce Atrophy and Promote Functional Repair in a Model of Stroke, Cell reports, 20 (2017) 1964-1977.

[46] N. Tajiri, D.M. Quach, Y. Kaneko, S. Wu, D. Lee, T. Lam, K.L. Hayama, T.G. Hazel, K. Johe, M.C. Wu, C.V. Borlongan, Behavioral and histopathological assessment of adult ischemic rat brains after intracerebral transplantation of NSI-566RSC cell lines, PLoS One, 9 (2014) e91408.

[47] K. Takahashi, T. Yasuhara, T. Shingo, K. Muraoka, M. Kameda, A. Takeuchi, A. Yano, K. Kurozumi, T. Agari, Y. Miyoshi, K. Kinugasa, I. Date, Embryonic neural stem cells transplanted in middle cerebral artery occlusion model of rats demonstrated potent therapeutic effects, compared to adult neural stem cells, Brain Res, 1234 (2008) 172-182.

[48] Y. Tang, J. Wang, X. Lin, L. Wang, B. Shao, K. Jin, Y. Wang, G.Y. Yang, Neural stem cell protects aged rat brain from ischemia-reperfusion injury through neurogenesis and angiogenesis, Journal of cerebral blood flow and metabolism : official journal of the International Society of Cerebral Blood Flow and Metabolism, 34 (2014) 1138-1147.

[49] J. Tatarishvili, K. Oki, E. Monni, P. Koch, T. Memanishvili, A.-M. Buga, V. Verma, A. Popa-Wagner, O. Bruestle, O. Lindvall, Z. Kokaia, Human induced pluripotent stem cells improve recovery in stroke-injured aged rats, Restor. Neurol. Neurosci., 32 (2014) 547-558.

[50] M.H. Theus, L. Wei, L. Cui, K. Francis, X. Hu, C. Keogh, S.P. Yu, In vitro hypoxic preconditioning of embryonic stem cells as a strategy of promoting cell survival and functional benefits after transplantation into the ischemic rat brain, Exp Neurol, 210 (2008) 656-670.

[51] J. Yamane, S. Ishibashi, M. Sakaguchi, T. Kuroiwa, Y. Kanemura, M. Nakamura, H. Miyoshi, K. Sawamoto, Y. Toyama, H. Mizusawa, H. Okano, Transplantation of human neural stem/progenitor cells overexpressing galectin-1 improves functional recovery from focal brain ischemia in the Mongolian gerbil, Mol Brain, 4 (2011) 35.

[52] T. Yang, K.S. Tsang, W.S. Poon, H.K. Ng, Neurotrophism of bone marrow stromal cells to embryonic stem cells: noncontact induction and transplantation to a mouse ischemic stroke model, Cell Transplant, 18 (2009) 391-404.

[53] H. Yao, M. Gao, J.H. Ma, M.Y. Zhang, S.W. Li, B.S. Wu, X.H. Nie, J. Jiao, H. Zhao, S.S. Wang, Y.Y. Yang, Y.S. Zhang, Y.L. Sun, M.S. Wicha, A.E. Chang, S.R. Gao, Q. Li, R.X. Xu, Transdifferentiation-Induced Neural Stem Cells Promote Recovery of Middle Cerebral Artery Stroke Rats, PLoS One, 10 (2015).

[54] T. Yuan, W. Liao, N.-H. Feng, Y.-L. Lou, X. Niu, A.-J. Zhang, Y. Wang, Z.-F. Deng, Human induced pluripotent stem cell-derived neural stem cells survive, migrate, differentiate, and improve neurologic function in a rat model of middle cerebral artery occlusion, Stem Cell Res. Ther., 4 (2013).

[55] F. Zhang, X.H. Duan, L.J. Lu, X. Zhang, M.W. Chen, J.J. Mao, M.H. Cao, J. Shen, In Vivo Long-Term Tracking of Neural Stem Cells Transplanted into an Acute Ischemic Stroke model with Reporter Gene-Based Bimodal MR and Optical Imaging, Cell Transplantation, 26 (2017) 1648-1662.

[56] G.L. Zhang, X.Y. Guo, L.K. Chen, B.Q. Li, B. Gu, H. Wang, G.J. Wu, J. Kong, W.H. Chen, Y.B. Yu, Interferon- Promotes Neuronal Repair by Transplanted Neural Stem Cells in Ischemic Rats, Stem Cells and Development, 27 (2018) 355-366.

[57] P. Zhang, J. Li, Y. Liu, X. Chen, Q. Kang, J. Zhao, W. Li, Human neural stem cell transplantation attenuates apoptosis and improves neurological functions after cerebral ischemia in rats, Acta Anaesthesiol Scand, 53 (2009) 1184-1191.

[58] T. Zhang, X. Yang, T. Liu, J. Shao, N. Fu, A. Yan, K. Geng, W. Xia, Adjudin-preconditioned neural stem cells enhance neuroprotection after ischemia reperfusion in mice, Stem cell research & therapy, 8 (2017) 248.

[59] Z.-h. Zhang, R.-z. Wang, R.-z. Wang, G.-l. Li, J.-j. Wei, Z.-j. Li, M. Feng, J. Kang, W.-c. Du, W.-b. Ma, Y.-n. Li, Y. Yang, Y.-g. Kong, Transplantation of neural stem cells modified by human neurotrophin-3 promotes functional recovery after transient focal cerebral ischemia in rats, Neurosci. Lett., 444 (2008) 227-230.

[60] Y. Zhao, S.T. Yao, S.J. Wang, Neural stem cell transplantation in the hippocampus of rats with cerebral ischemia/reperfusion injury Activation of the phosphatidylinositol-3 kinase/Akt pathway and increased brain-derived neurotrophic factor expression, Neural Regen. Res., 5 (2010) 1605-1610.

[61] J.M. Zhu, Y.Y. Zhao, S.D. Chen, W.H. Zhang, L. Lou, X. Jin, Functional Recovery after Transplantation of Neural Stem Cells Modified by Brain-derived Neurotrophic Factor in Rats with Cerebral Ischaemia, J. Int. Med. Res., 39 (2011) 488-498.

[62] W. Zhu, Y. Mao, Y. Zhao, L.F. Zhou, Y. Wang, J.H. Zhu, Y. Zhu, G.Y. Yang, Transplantation of vascular endothelial growth factor-transfected neural stem cells into the rat brain provides neuroprotection after transient focal cerebral ischemia, Neurosurgery, 57 (2005) 325-333; discussion 325-333.
