# Supplementary material for: Intraparenchymal Neural Stem/Progenitor Cell Transplantation for Ischemic Stroke Animals: A Meta-Analysis and Systematic Review
Source: Stem Cells Int. 2018 Oct 2;2018:4826407. doi: 10.1155/2018/4826407 (PMC6189667; doi:10.1155/2018/4826407)
Supplement: Supplementary 6 — Table S5: reasons for excluding related comparisons. [file 4826407.f6.docx]

Table S5. Reasons for excluding related comparisons

| Comparisons | Exclude reasons | |
| --- | --- | --- |
| mNSS |  |  |
| Chang, 2013 | After cell transplantation at 1 week post stroke, the score made significant difference compared with control group, and the difference appeared at every detection point. No blinding and randomization design. |  |
| Chang, 2013 | There was significant difference as early as 1 week post cell transplantation compared with control group, and the difference last for the whole period (8 weeks). No blinding and randomization design. |  |
| Kim, 2014 | There was significant difference as early as 3 days post cell transplantation compared with control group, and the difference last for the whole period (24 days). No blinding design. |  |
| Tang, 2014-2 | There was no difference among the 2 weeks for cell treated group. And the outcomes at 2nd week was worse than that of the 1st week. |  |
| Zhang, 2009 | There was significant difference as early as 1 week post cell transplantation compared with control group, and the difference last for the whole period. |  |
| Zhang, 2017-1 | There was significant difference as early as 1 week post cell transplantation compared with control group, and the difference last for the whole period. |  |
| Zhang, 2018 | The mNSS score ranged from 10 to less, and within a week, there was also a significant difference. |  |
| Rotarod test |  |  |
| Doeppner, 2015-1 | The cell transplantation did not improve performance on the rotarod. At the final detection point, the performance was worse evenly. |  |
| Doeppner, 2015-3 | The cell treated group show shorter maintaining time on the rotarod from the second week. |  |
| Ma, 2015 | There was no difference between vehicle and sham groups at the terminal time point. No randomization design. |  |
| Takahashi, 2008 | The function was improved at the first day post transplantation. And the values at this time point made no difference with the terminal time point (28 days). No blinding and randomization design. |  |
| Doeppner, 2017 | The score indicated amelioration alone the whole process. No randomization design. |  |
| Zhang, 2017-2 | For 120min ischemia/reperfusion injury, there has been a significant difference within a week. And the numbers of animals used were less than six. |  |
| Infarct volume reduction |  |  |
| Eckert, 2015 | The outcomes were calculated at the following day post stroke, with the numbers of animals used less than six. No blinding and randomization design. |  |
| Huang, 2014 | The outcomes were calculated at the following day post stroke, with the numbers of animals used less than six. No blinding and randomization design. |  |
| Lu, 2017 | No sham control and no blinding design. |  |
| Zhang, 2018 | For serve brain ischemia, there has been a significant difference within a week. And the numbers of animals used were less than six. |  |
